# *Bacillus velezensis* TB918 mitigates garlic dry rot disease by forming consortia with *Pseudomonas* in the rhizosphere and bulb

**DOI:** 10.3389/fmicb.2025.1567108

**Published:** 2025-04-15

**Authors:** Haowen Shi, Bingbing Sun, Beiying Sun, Xiuli Wang, Bing Li, Feng Wu, Tao Tian

**Affiliations:** ^1^Institute of Plant Protection, Tianjin Academy of Agricultural Sciences, Tianjin, China; ^2^Department of Geography, University College London, London, United Kingdom; ^3^Lanzhou Productivity Promoting Center, Gansu, China; ^4^Tianjin Agricultural Development Service Center, Tianjin, China; ^5^Institute of Vegetables, Tianjin Academy of Agricultural Sciences, Tianjin, China

**Keywords:** *Bacillus velezensis* TB918, *Fusarium*, garlic dry rot, biocontrol, rhizosphere microbiota

## Abstract

Garlic dry rot (GDR), primarily caused by *Fusarium proliferatum*, is a significant postharvest disease that leads to substantial economic losses. Our previous research demonstrated that supplementing *Bacillus*-based biocontrol formulations with sucrose could boost its efficiency in protecting plants by building a hostile rhizomicrobiome for destructive soil-borne pathogens. *B. velezensis* TB918, previously isolated from pepper rhizosphere soil, exhibited a strong *in vitro* antifungal effect on *Fusarium*. In this study, we conducted a field experiment to investigate the efficacy of *B. velezensis* TB918 in controlling GDR, and explored the changes in microbial communities in garlic plants and rhizosphere soil following the application of TB918 with or without sucrose supplementation. Using 16S rRNA and ITS amplicon sequencing, we found that the introduction of TB918 significantly increased the abundance of *Pseudomonas* in garlic rhizosphere, especially when combined with sucrose. Three *Pseudomonas* strains were isolated from garlic tissues and rhizosphere soil treated with TB918 and sucrose, among which the GP2 strain exhibited antagonistic effects against pathogen *ad planta*. Co-culture and colonization assays showed that TB918 facilitated the biofilm formation of *Pseudomonas* strain by forming consortia. Interestingly, the abundance of potentially non-pathogenic *Fusarium concentricum* also increased, suggesting a potential niche exclusion effect. Our results demonstrated that TB918 in combination with sucrose effectively reduced the incidence of GDR during storage. This study provides valuable insights into the use of biocontrol agents and sucrose to modulate the garlic microbial community and suppress soil-borne pathogens.

## Introduction

Garlic (*Allium sativum* L.) is an important horticultural crop that has a perennial bulb and has been cultivated worldwide in temperate zones. Currently, garlic is cultivated on 1.69 million hectares, and its production is more than 28 million tons worldwide (FAO-STAT, 2023). However, multiple diseases threaten the quality and yield of garlic. In particular, different postharvest diseases cause great economic losses during the processes of drying, storage, transportation, and marketing. Garlic dry rot (GDR) caused by *Fusarium proliferatum* is one of the most important postharvest diseases on the bulb, which has caused up to 30% of postharvest yield losses in recent years (Tonti et al., 2012; Gálvez and Palmero, 2022). The initial symptoms of dry rot include necrotic and centrally depressed spots on garlic bulbs, often with white mycelium developed. Symptoms are not obvious before peeling, but progress during the storage process, resulting in emptied sheaths, even under cold storage conditions at 4°C (Seefelder et al., 2002). During the growing season, pathogenic infections can be observed mainly on roots and basal plates. Severe infestations disrupt nutrient translocation and induce cellular necrosis, leading to significant mortality in garlic populations under sustained biotic pressure (Mondani et al., 2021). *F. proliferatum* can be detected in garlic bulbs during the early growth stages and healthy-looking garlic cloves, suggesting a potential seed transmission route (Mondani et al., 2020). Moreover, *F. proliferatum*, which has also been frequently isolated from garlic fields, can reduce clove germination, produce extensive seedling damping-off, and cause high disease severity of rotted roots/cloves (Gálvez and Palmero, 2022). Several plant-pathogenic strains within the genus *Fusarium* were found to produce various metabolites including fumonisins (FB1, FB2, and FB3) (Seefelder et al., 2002; Seefelder et al., 2004; Boonzaaijer et al., 2008; Tonti et al., 2017; Mondani et al., 2020), moniliformin (MON) (Marasas et al., 1986), beauvericin (BEA) (Plattner and Nelson, 1994; Logrieco et al., 1998), fusaric acid (FA) (Bacon et al., 1996), and fusaproliferin (FUP) (Ritieni et al., 1995), which threaten food safety seriously and have carcinogenic potential (Rheeder et al., 2002; Palmero et al., 2012; Cendoya et al., 2014). Therefore, it is impending to control this pathogen during the planting and postharvest storage of garlic.

Although GDR has been reported in multiple nations, research focusing on epidemiological analyses or agricultural management practices aiming at controlling GDR remains limited. Some studies have shown that certain chemical and physical treatments, such as benomyl (Dugan et al., 2007), carbendazim (Elshahawy et al., 2017), and thermotherapy (Palmero Llamas et al., 2013), are effective against *Fusarium* spp. in garlic, but these fungicides and treatments still have limitations or deficiencies in controlling GDR in the field (Gálvez Patón et al., 2017; López-Coria et al., 2023). However, Jo et al. (2021) used transcriptomic data to examine tissue-specific garlic microbiomes and to indicate the role of microbial communities in garlic health. They revealed that the microbial communities in garlic varied depending on the tissue types, although there was one dominant microorganism in each tested tissue. Moreover, the ability of biocontrol agents such as *Bacillus* and *Trichoderma* spp. to control *Fusarium* spp. *in vitro* has been tested in garlic crops (Mondani et al., 2021). Zhuang et al. (2021) reported that *Pseudomonas* is a key element in the garlic rhizosphere for the healthy growth of garlic. A synthetic community with six *Pseudomonas* strains isolated from the garlic rhizosphere showed an outstanding ability to promote growth. Otherwise, biocontrol agents have also been reported to manage pathogen invasion of plant roots and affect postharvest storage by regulating the rhizosphere microbiome (Yao et al., 2021). Although previous studies on biocontrol agents in garlic production have shown promising results, there is currently a lack of information regarding the changes in the microbial community structure and the interactions between bacteria and fungi.

Our previous study demonstrated that the root-secreted sucrose plays an important role in shaping the rhizosphere microbiota and establishing symbiosis between *Bacillus* and plant roots (Tian et al., 2020). Sucrose (1) induces solid surface motility and promotes root colonization by *Bacillus*, (2) selectively shapes the rhizomicrobiome by increasing the abundance of *Bacillus* and *Pseudomonas* species, which are known for their plant-beneficial traits, and (3) improves biological control efficacy of *B. subtilis* against soil-borne diseases (Tian et al., 2021). Our previous research showed that supplementing sucrose enhanced the biocontrol efficiency of *B. subtilis* 3610 by creating a rhizomicrobiome unfavorable to destructive soil - borne pathogens. These findings suggest a practical approach to boost the prevalence of beneficial *Bacillus* species in plant protection by manipulating sucrose availability.

*B. velezensis* TB918 was initially isolated from pepper rhizosphere and exhibited a strong *in vitro* antifungal effect on *Fusarium* spp. Given the potential of TB918 as a biocontrol agent and the positive role of sucrose in biocontrol, we hypothesized that the combination of TB918 and sucrose might effectively control GDR. We applied the TB918 strain and sucrose via root irrigation in a field experiment. The primary aim was to investigate the effects of these treatments on the incidence and severity of GDR. Additionally, we aimed to analyze the changes in the structure and diversity of the microbial community in garlic plants and rhizosphere soil following the application of these treatments. This study aims to provide insights into the potential of using biocontrol agents and sucrose to modulate the microbiome and suppress soil-borne pathogens in garlic cultivation.

## Materials and methods

### The strains, growth media, and construction of mutants

The *B. velezensis* TB918 strain was initially isolated from pepper (*Capsicum annuum*) rhizosphere soil in Tianjin, China, in 2011, and preserved at the Tianjin Academy of Agricultural Sciences. TB918 cells were cultured in Luria Bertani (LB) liquid media consisting of 10 g/L tryptone, 5 g/L yeast extract, and 10 g/L NaCl. The culture was shaken (150 rpm) overnight at 32°C, after which the concentration and activity of the bacteria were measured. The bacterial suspension was washed twice with physiological saline, and the concentration of the suspension was adjusted to 1 × 10^8^ CFU/mL. The TB918 suspension was mixed with sterilized 30% (v/v) glycerol at the ratio of 1:1 and stored at −80°C for long-term preservation.

The commercially available biocontrol agent *Paenibacillus polymyxa* KN-03 was selected based on its ability to reduce the growth of *Fusarium*. This biocontrol agent was provided by Wuhan Kernel Bio-tech Co., Ltd. (Wuhan, China) and the concentration of the active ingredient was 5 × 10^8^ CFU/mL according to the manufacturer.

Because *B. velezensis* TB918 was not be genetically manipulated yet, another closely related species, *B. subtilis* 3610, was used for the root colonization test. The construction of *B. subtilis* 3610 mKate2 mutant strain was performed as previously described (Tian et al., 2021). Briefly, the mKate2 gene, encoding a far-red fluorescent protein, was amplified via PCR with flanking sequences homologous to the target locus (*amyE*). A temperature-sensitive plasmid pDG1661 containing a spectinomycin resistance cassette (*specR*) was utilized as the integration vector. The plasmid was linearized and fused with the mKate2 fragment using Gibson Assembly (NEB). The recombinant plasmid was transformed into *B. subtilis* 3610 via electroporation, followed by selection at 30°C on LB agar supplemented with spectinomycin. Positive integrants were verified by colony PCR. To eliminate the plasmid, a temperature shift to 42°C was applied to induce plasmid loss. Successful markerless replacement was confirmed by PCR screening and sequencing. Fluorescence microscopy and spectral analysis were conducted to validate mKate2 expression.

The construction of GFP-labeled *P. fluorescens* mutants could be achieved as previously described (Lai et al., 2022)‌. A broad-host-range plasmid pBBR1-MCS5 carrying the *gfp* gene under a constitutive promoter *Ptac* and a kanamycin resistance marker was first assembled. For chromosomal integration, homologous arms flanking the target locus (*attB*) were designed and amplified via PCR with primers containing restriction enzyme sites (EcoRI/HindIII)‌. The *gfp* cassette and homologous arms were ligated into the plasmid backbone using Gibson Assembly (NEB)‌. The recombinant plasmid was introduced into *P. fluorescens* via electroporation‌. Transformants were selected on LB plate containing kanamycin and verified by colony PCR targeting the *gfp*-homology junctions‌. To enhance screening efficiency, GFP-positive colonies were identified using blue light excitation (488 nm) and fluorescence microscopy‌. Plasmid stability and markerless integration were confirmed through sequential subculturing in non-selective media and PCR sequencing‌.

### Study site description and experimental design

The study site was located at the Modern Agricultural Technology Innovation Base (Wuqing District, Tianjin, 39°42′88″N, 117°10′76″E) in the Tianjin Academy of Agricultural Sciences, where nearly 10 hectares of fields have been managed under controlled conditions for 10 years. For this study, a garlic plantation covering 1,200 m^2^ was established in February 2020 and the field management was carried out continuously in accordance with the standard production procedure. The selected garlic cultivar was “Liu Ban Hong,” a common locally cultivated variety. The garlic cultivation management was the same as the field standardized management method in the North China Plain. Soil was deep-plowed (30–40 cm) and supplemented with 8,000 kg/ha fully decomposed organic manure and 200–300 kg/ha compound fertilizer (N-P_2_O_5_-K_2_O = 15–15-15) as basal dressing. Sowing was conducted in early spring (March) to avoid winter frost damage, ensuring soil temperature above 3°C. Garlic row spacing was 30–40 cm, plant spacing 10–15 cm, planting depth 3–4 cm with clove orientation pointed end upwards to optimize emergence. Sowing density was 40,000–50,000 plants/ha (optimized for medium-late maturing varieties). Soil moisture was maintained at 60–70% field capacity. Critical phases include post-sowing (to ensure germination), and bulb expansion stages (to prevent drought stress). Pesticide were applied to ensure that there were no threats‌ by pests and weeds during the growth process and to avoid influencing the corresponding agronomic traits. Pendimethalin (33% EC) was applied as a ‌soil treatment‌ once ‌post-sowing‌ at a rate of ‌2.2–3.0 L/ha to control ‌annual grass and broadleaf weeds‌. Imidacloprid (10% WP) was used as a foliar spray at a 2,500-fold dilution (300 g/750 L water per ha) during aphid infestations, with applications repeated at 7-day intervals for up to ‌3 consecutive cycles.

To explore efficient and safe measures for GDR control, the *B. velezensis* TB918 strain, sucrose, and the commercial bioagent *P. polymyxa* were applied 1 month after of field planting in 2020 and 2021. The experimental treatments included (1) CK (irrigation water), (2) inoculation with *B. velezensis* TB918, (3) TB918 with sucrose supplementation, and (4) the commercial bioagent *P. polymyxa*. Both the *B. velezensis* TB918 and *P. polymyxa* bioagents were applied at 75 L/ha after 100-fold dilution with irrigation water. The supplementation of sucrose was 0.5% (w/v) in the final formulation. The water consumption of the CK group was consistent with the amount of irrigation water applied to the TB918 treatment group. The garlic roots were treated with the bioagents and water twice by drip irrigation at a two-week interval. This field plot was surveyed based on the history of GDR in the past 2 years. The field was divided into four blocks (for four treatments) randomly with three equal-sized plots (3 × 10 = 30 m^2^ for each plot) spaced 50 cm apart.

### Sample collection

The margin bulk and rhizosphere soil samples were collected when garlic was harvested during the mature period in 2021. For rhizosphere soil samples, the garlic plant was excavated with a spade, and loosely adhering soil was removed by vigorous shaking. The tightly adhering soil around the garlic root, regarded as rhizosphere soil, was placed in a sterilized 50 mL Falcon tube, and visible garlic tissues and roots were removed carefully from each sample. Each rhizosphere soil sample was a mixture of approximately 50 g from 20 randomly chosen garlic plants without obvious disease symptoms. For the margin bulk soil samples, which were located at the edge of the field 20 cm away from plants in non-cultivated areas, the upper soil layer was removed by a spade, and the soil from a depth of 10 cm below the ground was collected with a sterilized scoop and placed in a sterilized 50 mL Falcon tube. Each margin bulk soil sample was a mixture from 5 random margin positions around the field. Three replicate margin and rhizosphere soil samples of the mixture and all related garlic plants were placed in an ice box and transferred to the laboratory within 1 h for further testing. In parallel, the same amounts of garlic plants subjected to different treatments were collected by the above method for storage tests to assess the disease incidence rate and severity.

The garlic leaves and roots were detached, and the bulb was selected for tissue separation in a sterile environment. The surface soil of all garlic bulbs was removed with a sterilized brush, and the plants were separated with sterilized tweezers into outer sheaths (cover entire bulb, n = 20/treatment), inner sheaths (cover each clove, n = 100/treatment) and garlic cloves (n = 100/treatment) and collected individually. These tissue samples were all cut into small pieces with sterile scissors. All samples were stored at −80°C until further use.

### Postharvest storage, disease incidence statistics, and disease severity assessment

After harvest, the garlic plant samples for the storage test were sun-dried on field ground for 20 days with coverage in case of rain. Afterwards, garlic plants were taken to cold rooms at −4°C and 90% relative humidity for 6 months of storage according to the standard storage conditions and mean storage time.

After postharvest storage, the incidence of GDR on the bulbs was investigated in 2020 and 2021. The diseased plants were selected and assessed for the presence and severity of dry rot. Disease incidence (%) = number of infected plants × 100/total number of plants in each replicate.

We graded GDR disease severity according to the extent of clove rot: 0 = asymptomatic; 10% = small lesions on cloves; 30% = small depressed brown spots; 60% = one or more extended depressed brown spots; 85% = extended lesions or spots on one or more surfaces on cloves with a dehydrated appearance; and 100% = completely rotted cloves with a dehydrated appearance and visible white mycelium. The disease severity index was calculated for each replicate of 10 cloves according to the following formula (Mondani et al., 2020):


$$\begin{array}{l} \mathrm{The disease severity index}\\ =\frac{\left(10\%\times n1\right)+\left(30\%\times n2\right)+\left(60\%\times n3\right)+\left(85\%\times n4\right)+\left(100\%\times n5\right)}{10}\text{.} \end{array}$$

**Figure 1:**
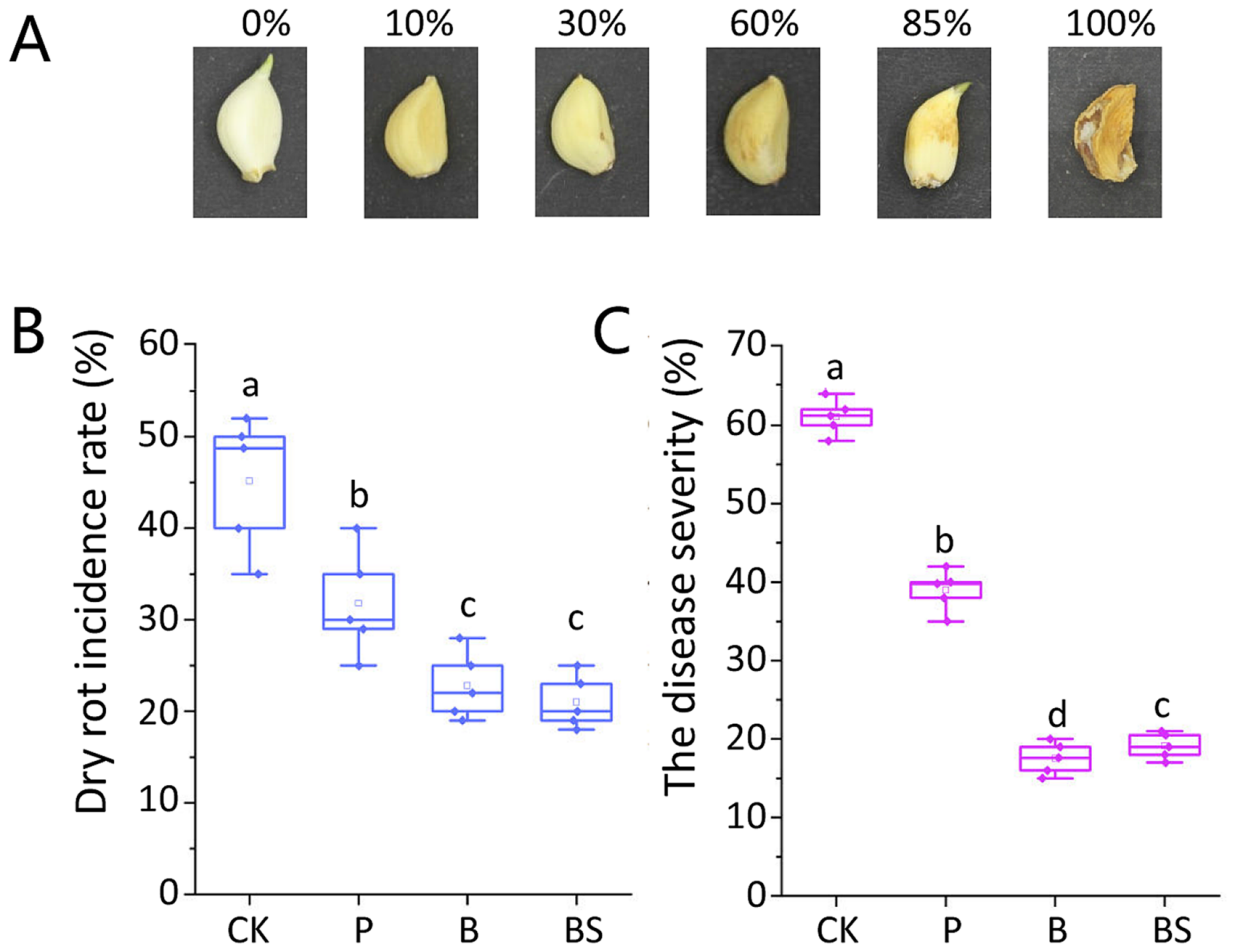
Garlic dry rot (GDR) typical symptoms on cloves and effects of biocontrol agents against GDR. **(A)** Symptoms on garlic cloves were observed 6 months after harvest. The GDR disease severity was graded into six classes: 0 = asymptomatic; 10% = small lesions on cloves; 30% = small depressed brown spots; 60% = one or more extended depressed brown spots; 85% = extended lesions or spots on one or more surfaces on cloves with dehydrated appearance; 100% = completely rotted cloves with dehydrated appearance and visible white mycelium. The bio-agent *B. velezensis* TB918 and paired with sucrose significantly reduced the incidence rate **(B)** and disease severity **(C)** of GDR. CK: irrigation water, P: commercial biocontrol agent *P. polymyxa*, B: *B. velezensis* TB918, BS: *B. velezensis* TB918 and sucrose. Both indexes were calculated as the mean of 10 cloves of each treatment and five test replicates. The bars represent the standard error. The letters above the columns indicate statistically significant differences based on the index using the Student’s *t*-test (*p* < 0.05).

Where 10, 30, 60, 85, and 100% represent disease severity; n1, n2, n3, n4, and n5 represent the number of cloves with 10, 30, 60, 85, and 100% disease severity, respectively; and 10 represents the number of cloves in each replicate. Typical symptoms of GDR are shown in Figure 1A. The value calculated by this formula represents a comprehensive disease index for the GDR.

### Strain isolation and verification

Infected garlic bulbs were sampled to isolate and determine the identity of the disease-causing microorganism. Symptomatic bulbs were surface sterilized with 1% NaClO for 1 min, dipped in 75% ethanol for 5 min and rinsed three times with sterile distilled water. Small pieces of the inner decayed tissue were removed and cultured on potato dextrose agar (PDA) at 28°C for 7 days. Four white colonies grew on PDA and then they became dark-gray to blackish-gray mycelium. All the fungal strains were purified by the monosporic isolation method (Balmas et al., 2010). The media used in this study include PDA medium and PSA (potato sucrose agar) medium. PDA medium consists of 20% (w/v) potato, 2% (w/v) glucose, and 1.5% (w/v) agar. PSA was prepared from PDA, replacing glucose with 2% (w/v) sucrose.

To complete Koch’s postulates, surface-sterilized healthy garlic bulbs were inoculated with four fungal isolates. A 1.0-mL sterile syringe was used to inject 50 μL of a 10^6^ conidia/ml suspension with sterile ddH_2_O into each of five healthy bulbs. As a control, garlic bulbs were treated with sterile distilled water. The inoculated and control bulbs were incubated at 28°C for 14 days. The bulbs inoculated with *F. proliferatum* GCF4 showed symptoms of water soaking, and the tissues were brown and soft throughout the bulb at 14 days. All other isolates were nearly symptomless. The experiment was performed in triplicates, with consistent results. No disease symptoms were observed in the control group. *F. proliferatum* was reisolated from the symptomatic garlic bulbs and re-identified based on colony and sporangia morphology and ITS sequence.

To isolate cooperating *Pseudomonas* strains, fresh garlic tissues and rhizosphere soil inoculated with strain TB918 and sucrose were collected and suspended in PBS buffer and vortexed vigorously. Dilution series were plated on LB medium with 1.5% agar and incubated at 28°C for 24 h. Three dominant single colonies were picked and purified two times. The purified colonies were showed fluorescent pigments on King’s B medium (King et al., 1954). Glycerol stocks were prepared by suspending the isolates (grown in LB broth under shaking conditions) in sterilized 30% (v/v) glycerol at the ratio of 1:1 and stored at −80°C.

The *in vitro* antagonistic activities of *Pseudomonas* strains against *Fusarium* isolates were tested. Four *Fusarium* isolates were grown on PDA plates for 7 days at 25°C in the dark. A fungal plug of 5 mm diameter was placed at the center of a 90 mm PDA plates. The plates were incubated at 25°C until the growing fungal colony without bacterial inoculation reached a radius of 15 mm. The tested *Pseudomonas* strains and *B. velezensis* TB918 were each pre-cultured overnight in LB liquid medium, and 5 mL of each culture was centrifuged at 5,000 rpm for 5 min. The pellets were resuspended in sterile ddH_2_O to an optical density (OD) of 1 at 600 nm. One microliter droplets of the bacteria cell suspension was inoculated onto the surface of the fungal PDA plates 15 mm from the plate periphery. After this, all plates were wrapped with parafilm incubated at 28°C for 3 days and observed for the inhibition of the *Fusarium* strains.

### DNA extraction and PCR amplification

After harvesting, we extracted total DNA of each samples (0.5 g) from the outer sheaths, inner sheaths, garlic cloves, rhizosphere and margin soil. Microbial community genomic DNA was extracted and purified from soil samples using the E.Z.N.A.^®^ Soil DNA Kit (Omega Bio-tek, Norcross, GA, United States) and from garlic tissue samples using the FastPure^®^DNA Isolation Kit (Vazyme Biotech Co., Ltd., Nanjing, China) according to the manufacturer’s instructions. The DNA extract was checked on a 1% agarose gel, and the DNA concentration and purity were determined with a NanoDrop 2000 UV–vis spectrophotometer (Thermo Scientific, Wilmington, United States).

The PCR mixtures contained 5 × *TransStart* FastPfu buffer 4 μL, 2.5 mM dNTPs 2 μL, forward primer (5 μM) 0.8 μL, reverse primer (5 μM) 0.8 μL, *TransStart* FastPfu DNA Polymerase 0.4 μL, template DNA 10 ng, and finally ddH_2_O up to 20 μL. The hypervariable region V5-V7 of the bacterial 16S rRNA gene was amplified with the primer pair 799F (5′-AACMGGATTAGATACCCKG-3′) and 1193R (5′-ACGTCATCCCCACCTTCC-3′) (Mei et al., 2019). The hypervariable region ITS1-ITS2 of the fungal ITS sequence was amplified with the primer pair ITS1F (5′-CTTGGTCATTTAGAGGAAGTAA-3′) and ITS2R (5′-GCTGCGTTCTTCATCGATGC-3′) (Sang et al., 2020). The PCR conditions were as follows: initial denaturation at 95°C for 3 min, followed by 27 cycles of denaturing at 95°C for 30 s, annealing at 55°C for 30 s and extension at 72°Cfor 45 s, and a single extension at 72°C for 10 min, and end at 4°C. PCR reactions were performed in technical triplicates. The PCR product was extracted from 2% agarose gel and purified using an AxyPrep DNA Gel Extraction Kit (Axygen Biosciences, Union City, CA, United States) according to the manufacturer’s instructions and quantified using a Quantus™ Fluorometer (Promega, United States).

### Illumina MiSeq sequencing

The complete high-throughput gene sequencing of the purified amplicons was performed on the Illumina MiSeq platform (Majorbio BioPharm Technology Co., Ltd., Shanghai, China). The data were analyzed on the free online platform Majorbio Cloud Platform.^1^

The microbial communities of all samples were subjected to hierarchical clustering analysis using the Unweighted PairGroup Method with Arithmetic Mean (UPGMA). To determine the similarity and overlap of the species (such as OTU) composition of the environmental samples, OTUs with a similarity level of 97% were selected for analysis. According to the statistical analysis results, the Chao, ACE, Shannon, Simpson, and Coverage alpha indices reflect the richness and diversity of microbial communities, and corresponding rarefaction curves were analyzed with Mothur (v.1.30.1).^2^ The dynamic changes in the microbial community were classified at the phylum and genus levels by statistical analysis via R software (version 3.3.1).

### Phylogenetic analysis of the isolated strain

For three *Pseudomonas* isolates, bacterial DNA was extracted with the Bacterial Genome Extraction Kit (TIANGEN, China). The 16S rRNA gene was amplified with the primers 27F (5′-AGAGTTTGATCCTGGCTCAG-3′) and 1492R (5′-GGTTACCTTGTTACPGACTT-3′). The *gyrB* gene was amplified with the primers gyrB-F (5′-AGCATYAARGTGCTGAARGG-3′) and gyrB-R (5′-GGTCATGATGATGATGTTGTG-3′) (Huang et al., 2023). Fungal genomic DNA was extracted using Fungal DNA Extraction Kit (TianGen, China). For four *Fusarium* isolates, the ITS sequence was amplified with the primers ITS1 (5′-TCCGTAGGTGAACCTGCGG-3′) and ITS4 (5′-TCCTCCGCTTATTGATATGC-3′). The RPB2 gene was amplified with the primers RPB2-5F (5′-GAGTTCAAGATYTTCTCKGATGC-3′) and RPB2-7R (5′-GAATRTTGGCCATGGTRTCCAT-3′) (Yang et al., 2023). The PCR products were sequenced and blasted against the NCBI database (Johnson et al., 2008). A phylogenetic tree was established with Mega 7.0 software using the Neighbor-joining method.

### Biofilm formation and biomass quantification

In this study, biofilm refers to the aggregated growth of bacteria on a surface, which is composed of cells and extracellular polymeric substances (EPS). Colonies of *B. velezensis* TB918 and *Pseudomonas* strains were initially inoculated into 5 mL of MSgg liquid culture medium (5 mM potassium phosphate buffer pH 7, 100 mM MOPS pH 7, 2 mM MgCl_2_, 700 μM CaCl_2_, 50 μM MnCl_2_, 50 μM FeCl_3_, 1 μM ZnCl_2_, 2 μM thiamine, 0.5% glycerol, 0.5% glutamate, 50 μg/mL tryptophan, 50 μg/mL phenylalanine), and incubated at 28°C and 200 rpm until an OD_600_ of 0.6 was reached. To observe pellicle biofilm formation, an equal volume of *Bacillus* and *Pseudomonas* strains were mixed at an OD_600_ of 0.001, then 20 μL of the initial inoculum was cultivated in a 24-well microtiter plate (Corning) containing 2 mL MSgg liquid medium in each well. The microtiter plates were incubated statically at 28°C for 72 h. Then photos were taken to record the biofilm phenotypes. For each tested strain, three replicates were included.

The biomass quantification assay was modified from a method originally developed by (Sun et al., 2022). Pellicle biofilm was cultivated in 24-well microtiter plates (Corning) with 100 μm Sterile Nylon Mesh Cell Strainers in each well. Two milliliter aliquots of MSgg liquid medium and 20 μL of the initial inoculum were added. Six independent replicated wells were used for each tested strain. To allow the pellicle to grow on top of the nylon mesh cell strainer, the plates were incubated for 72 h at 28°C statically. The cell strainer was taken out and removed visible drops with paper. The dry weight was measured by drying the pellicle with the oven at 50°C for 12 h. Assays were done in triplicate.

### Laser scanning confocal microscope

The garlic cloves were surface sterilized by a 30 s soak in 75% (v/v) ethanol, followed by 5 min in sodium hypochloride (10% active chlorine) and by three subsequent wash steps with sterile water for 10 min each. Sterilized cloves were then aseptically positioned on a five-layer matrix of sterile glass beads (5 mm diameter) within an autoclaved 500-mL glass beaker, ensuring basal plate contact. The system was filled with sterile ddH_2_O to submerge the lower third of the cloves. Cultivation proceeded under controlled conditions (16 h light/8 h dark cycle at 22°C), with sterile ddH_2_O replenishment every 3 days. After 6 days of incubation, 5 mL overnight cultivated cell suspension (1.0 × 10^8^ CFU/mL) form *B. subtilis* 3610 mKate2 and *P. fluorescens* GFP mutants were inoculated individually or in combination via pipetting onto the root. After another 2 days of incubation, roots of seedlings were taken out and rinsed with sterilized water. Then 1 cm root ripening zone for each sample was taken and quickly stored in the sterile Eppendorf tube for use.

Collected root samples were mounted on microscope slides and were directly observed under the laser confocal microscope (Leica TCS SP8) at the excitation wavelength of 488 and 561 nm. All images were taken at the same exposure time and processed identically. The images were acquired using LAS X 4.1.1 and exported as tiff files. Each image is a representative of at least 10 root colonization assays performed in three independent experiments.

### Cell recovery counting

One milliliter sterile ddH_2_O was added to a 1.5 mL Eppendorf tube containing a 1 cm preprocessed root segment, followed by vortex mixing at maximum speed for 10 min. The resultant suspension was subjected to a serial dilution series (10^−2^ to 10^−4^) using sterile ddH_2_O. Aliquots (100 μL) from each dilution gradient were aseptically spread onto LB agar plates supplemented with the appropriate antibiotic. Plates were incubated at 28/32°C overnight. Colony-forming units (CFU) per millimeter of root tissue were calculated. The experiment was repeated three times with 10 root samples per replicate.

### Data and statistical analysis

Operational taxonomic units (OTUs) with a 97% similarity cutoff were clustered using UPARSE (version 7.1)^3^ (Edgar, 2013), and chimeric sequences were identified and removed. The alpha diversity of the microbial communities was estimated with QIIME2.^4^ Principal coordinate analysis (PCoA) using Bray-Curtis matrices was performed with the R package ade4 (v1.7.15), and analysis of similarities (ANOSIM) performed using the “vegan” package (v.2.5.4). Co-occurrence network analysis was conducted to characterize the complexity and modularity of microbial networks (Banerjee et al., 2018). The co-occurrence network was constructed based on Spearman’s significant correlation coefficient (|*r*| > 0.5, *p* < 0.05) in this study. The topology parameters, including node number, edge number, average degree and the ratio of positive to negative links, were calculated using Gephi (version 0.9.2) (Bastian et al., 2009). All the networks were visualized by using Cytoscape v3.8.0 (Shannon et al., 2003).

Disease incidence and severity index data were subjected to one-way analysis of variance using SPSS version 20.0 (SPSS Inc., Chicago, IL, United States). The letters above the columns indicate statistically significant differences based on Student’s *t*-test (*p* < 0.05). All error bars show the mean and standard deviation (SD). Each experimental treatment had three biological replicates. The microbiome difference analysis was performed using the Kruskal–Wallis one-way analysis of variance, which uses the rank sum of multiple samples to infer whether each sample represents the whole population. We used the kruskal.test package command via R software (version 3.3.1) to compare the relative abundance between different groups and to correct the test hypothesis.

## Results

### *B. velezensis TB918* significantly reduced the GDR incidence rate and disease severity

The GDR incidence rate was determined and the disease severity was calculated based on visible symptoms on garlic cloves after the 6-month storage according to the disease severity classes (Figure 1). The highest incidence rate of 45.2% was observed in the CK (irrigation water) garlic treatment, for which the mean disease severity reached 61.2%. Compared with the control group (CK), after inoculation with the biocontrol agents, the incidence rate and the disease severity were reduced significantly. The GDR incidence rate was similar between the samples using TB918 without or with sucrose addition (22.8% for B and 21.0% for BS). However, with TB918 and sucrose addition, the GDR severity (20.5%) was slightly greater than that of the sample with TB918 only (17.6%). As for commercial biocontrol agent *P. polymyxa*, its incidence rate (31.8%) and disease severity (39.8%) were significantly lower than those of CK but greater than those of TB918. Based on these results, we confirmed that compared with the CK treatment, all the biocontrol treatments significantly reduced the GDR incidence rate and disease severity.

### The microbial diversity and richness of garlic bulb-associated microbiota varies in different groups

To explore the impact of adding biocontrol agents on microbial diversity, we collected margin soil and field garlic samples with the commercial biocontrol agent, TB918 (with or without sucrose) and water (CK) addition. Three different garlic bulb tissues and rhizosphere soil were used for bacterial 16S rRNA (ribosomal RNA, V5-V7 region) analysis and ITS (internal transcribed spacer, ITS1-ITS2 region) analysis specifically for fungi. The sequencing results revealed 2,990,991 high-quality bacterial 16S tags (58,646 average tags per sample), and a total of 2,584 bacterial operational taxonomic units (OTUs) (Supplementary Table S1). Moreover, we acquired 5,066,192 high-qualities fungal ITS tags (99,337 average tags per sample), from which the clustering annotations totaled 1,984 OTUs (Supplementary Table S2).

After quality control, 538,662 16S sequences and 3,163,428 ITS sequences were available for further bacterial and fungal analysis, respectively. The mean lengths of both the 16S and ITS sequences were consistent with the expected PCR results. The community coverage of all the samples was greater than 0.966, further proving that most of the sequences contained in the garlic rhizosphere soil and tissues had been detected. The rank-abundance and rarefaction curves showed that the diversity of bacteria and fungi in the rhizosphere soil groups was obviously greater than that in the garlic tissue groups (Supplementary Figures S1, S2). Moreover, all rarefaction curves reached a plateau period proving that the sequencing depth was suitable.

**Figure 2:**
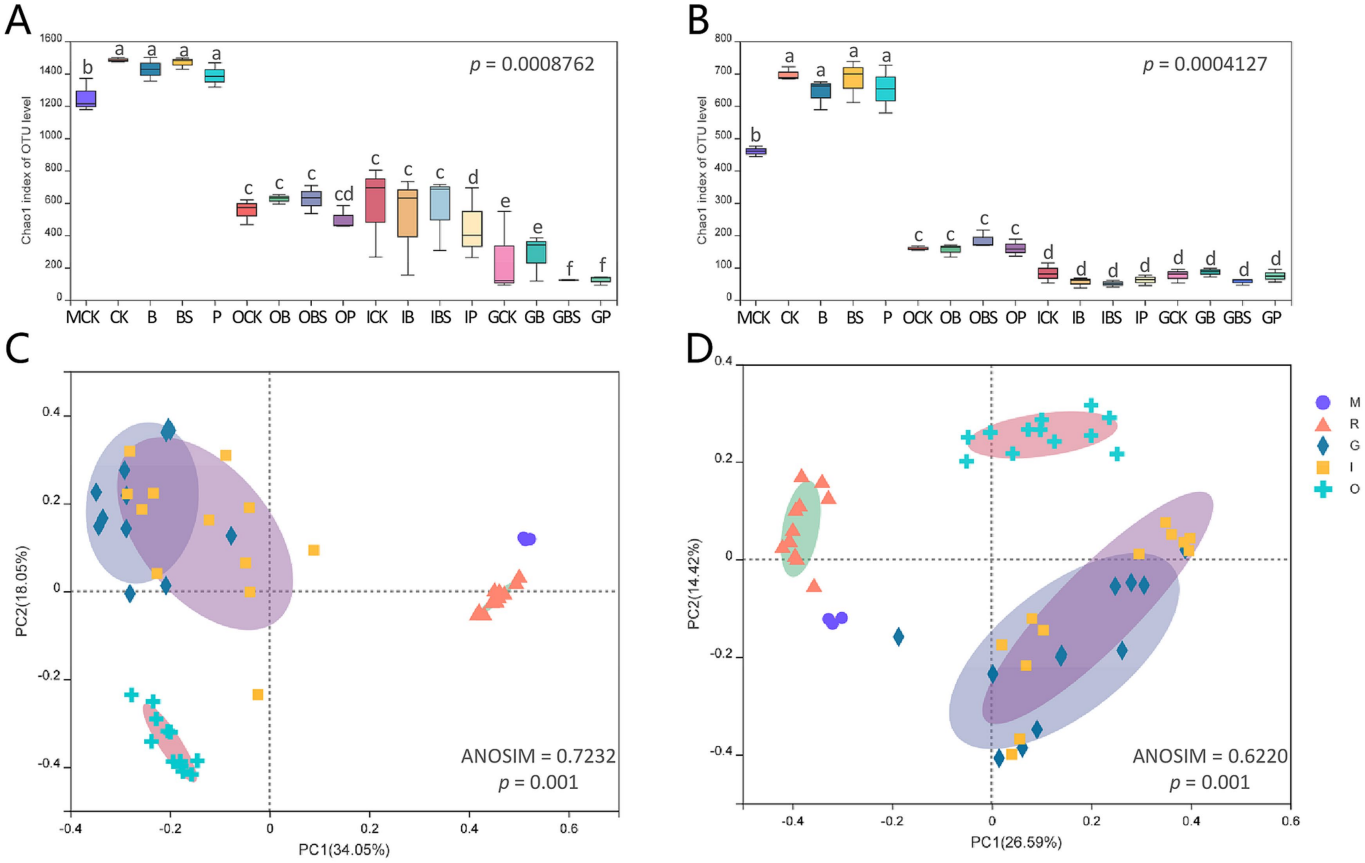
Diversity of microbiota associated with garlic bulb inoculating biocontrol agents. Alpha diversity of bacteria **(A)** and fungi **(B)** in different garlic bulb tissues and rhizosphere soil after inoculating biocontrol agents. CK (irrigation water), B (*B. velezensis* TB918), BS (*B. velezensis* TB918 and sucrose) and P (commercial biocontrol agent *P. polymyxa*) depict rhizosphere soil samples and different treatments. M (margin soil), G (garlic cloves), I (inner sheaths) and O (outer sheaths) depict different sampling locations. PCoA analysis of bacterial **(C)** and fungal **(D)** community structure in different garlic bulb tissues and rhizosphere soil, based on unweighted UniFrac distance. Samples were clustered into five groups: M (purple circle): margin soil group, R (orange triangle): rhizosphere soil group, G (cyan diamond): garlic cloves group, I (yellow square): inner sheaths group, O (blue cross): outer sheaths group.

The alpha diversity represents the richness and diversity of the microbial community. The Chao 1 and ACE indices are used to evaluate community richness, whereas the Shannon and Simpson indices reflect community diversity. The alpha indices and coverage were shown in Supplementary Tables S3, S4. The Chao 1 and ACE indices of rhizosphere soil for both bacteria (Chao 1: 1387.7–1483.5; ACE: 1391.2–1477.6) and fungi (Chao 1: 641.1–697; ACE: 649.9–709.8) were at the highest level among all samples, and were slightly greater than those of field edge margin soil (MCK) (Figures 2A,B). In addition, Chao 1 and ACE indices were highest in rhizosphere, followed by outer sheaths, followed by inner sheaths, and lowest in cloves.

Principal coordinate analysis (PCoA) (Figures 2C,D) revealed that the microbial communities identified in this study were clustered into five groups: the margin soil group (M), rhizosphere soil group (R), garlic clove group (G), inner sheath group (I) and outer sheath group (O). The structural difference in the microbial community between groups was greater than that within groups at different sample locations for both bacteria (ANOSIM = 0.7323, *p* = 0.001) and fungi (ANOSIM = 0.6220, *p* = 0.001), and it was suitable for the grouping scheme. The microbial communities in the margin and rhizosphere soil samples were separated from the garlic tissue samples along principal coordinate axis 1 (PC 1), which represented the greatest amount of variation (34.05% for bacteria, 26.59% for fungi). For both fungi and bacteria, the M, R, and O groups, which represented the distinct microbial communities, could be clearly separated. In addition, spatial proximity in the PCoA plot indicates similar community compositions in G and I groups.

### *B. velezensis* TB918 increased the abundance of *Pseudomonas* in rhizosphere bacterial community

**Figure 3:**
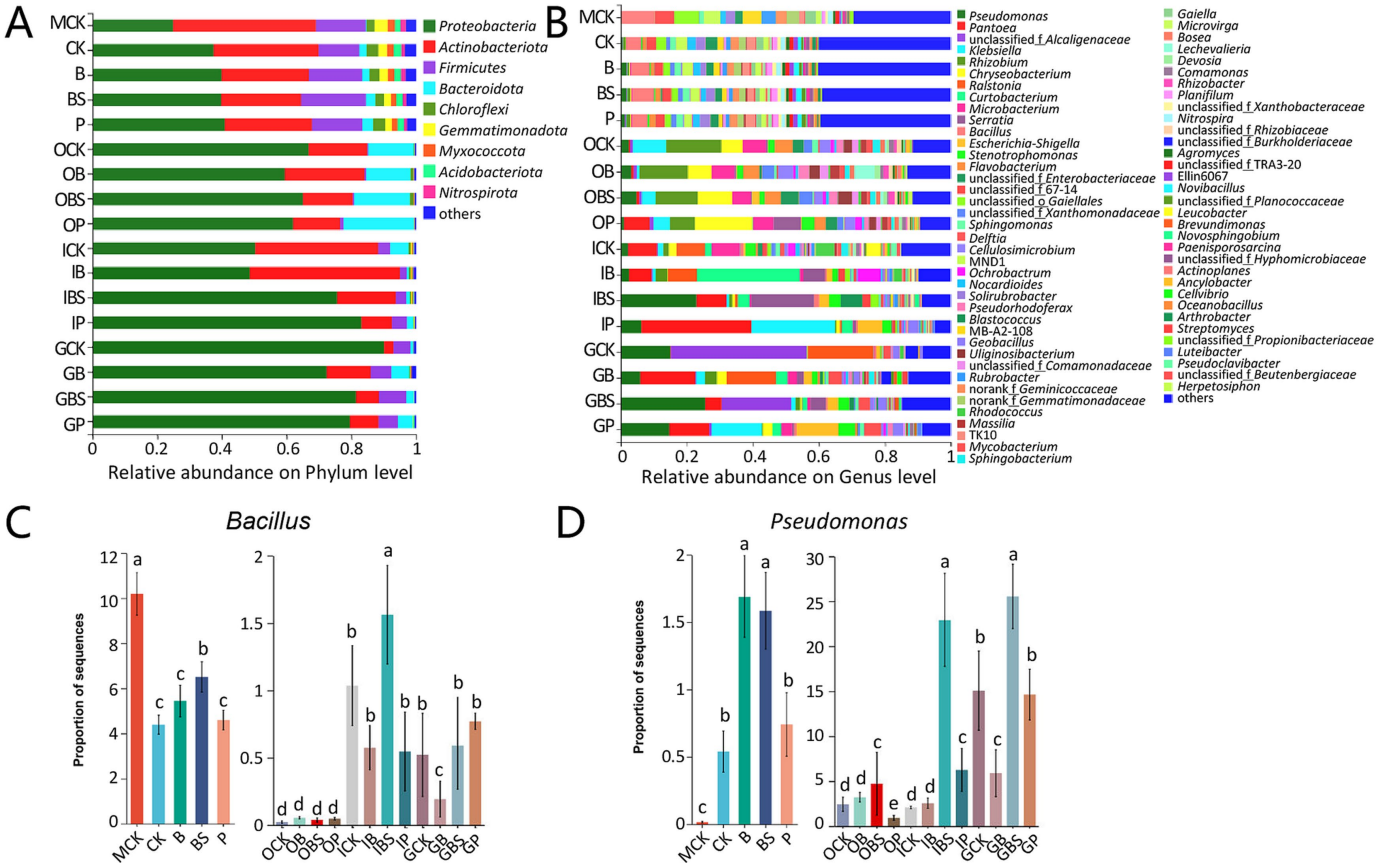
The bacterial community composition of garlic tissues and rhizosphere. Composition of different bacterial communities at the phylum **(A)** and genus **(B)** level. The relative abundance of different bacterial phylum or genus within the different communities is shown in different colors. Others: the merging of taxa with the relative abundance less than 0.01. The difference in the abundance of *Bacillus* **(C)** and *Pseudomonas* **(D)** based on Kruskal-Wallis H test (*p* < 0.05). Bars with different letters are significantly different (*p* < 0.05). CK (irrigation water), B (*B. velezensis* TB918), BS (*B. velezensis* TB918 and sucrose) and P (commercial biocontrol agent *P. polymyxa*) depict rhizosphere soil samples and different treatments. M (margin soil), G (garlic cloves), I (inner sheaths), and O (outer sheaths) depict different sampling locations.

The top five phyla in the whole dataset were *Proteobacteria*, *Actinobacteriota*, *Firmicutes*, *Bacteroidota*, and *Chloroflexi*, accounting for more than 96% of the entire microbial communities (Figure 3A; Supplementary Figure S3). The relative abundances of *Proteobacteria* were higher in garlic tissues, while that of *Actinobacteriota* was lower in garlic cloves and that of *Firmicutes* was lower in outer sheaths, compared with that in rhizosphere or margin soils.

At the genus level, further analysis revealed that distinct microbial taxa were enriched in different niches (Figure 3B). The relative abundance of *Bacillus* in rhizosphere did not significantly increase (4.39–5.45%) after the addition of TB918 or the commercial biocontrol agent, but it increased to 6.51% following sucrose supplementation. Our previous study also demonstrated that sucrose was indeed critical for *Bacillus* species to promote rhizosphere colonization (Tian et al., 2021). Notably, the compositions of the microbial communities in the margin (MCK) and rhizosphere (R) soils were similar at the phylum and genus levels, but there were significant structural differences between them, as evidenced by the PCoA results (Figure 2).

Though the emergence of TB918 did not affect the composition of the conserved microbial community at the phylum or genus level, the relative low abundance of *Pseudomonas* (CK: 0.54%) was higher in the rhizosphere (B: 1.69%) with the application of *Bacillus*, and this result was consistent with the analysis at the phylum level (Figures 3C,D). Interestingly, for the samples from the soil, the relative abundance of *Bacillus* at the margin reached 10.19%, which was obviously higher than that in the rhizosphere, possibly due to the stress resistance advantage of *Bacillus* against poor water and fertilizer conditions, lack of vegetation or frequent application of herbicides at the margin of the garlic field. However, the proportions of *Bacillus* in the rhizosphere were obviously higher than those in garlic tissues, but the trends in the proportions of *Pseudomonas* were exactly contrarious.

Moreover, the abundance of *Bacillus* was at the highest level but the abundance of *Pseudomonas* (0.01%) was at the lowest level in MCK (Figures 3C,D) In addition, the relative abundance of *Bacillus* in the inner sheath and garlic cloves is higher than that in the outer sheath. By comparing the community structure in different niches, it can be seen that sucrose addition could better induce an increase in the relative abundance of *Bacillus* and *Pseudomonas* in the inner sheath.

### *Fusarium* is affected by *B. velezensis* TB918 treatments in fungal community

**Figure 4:**
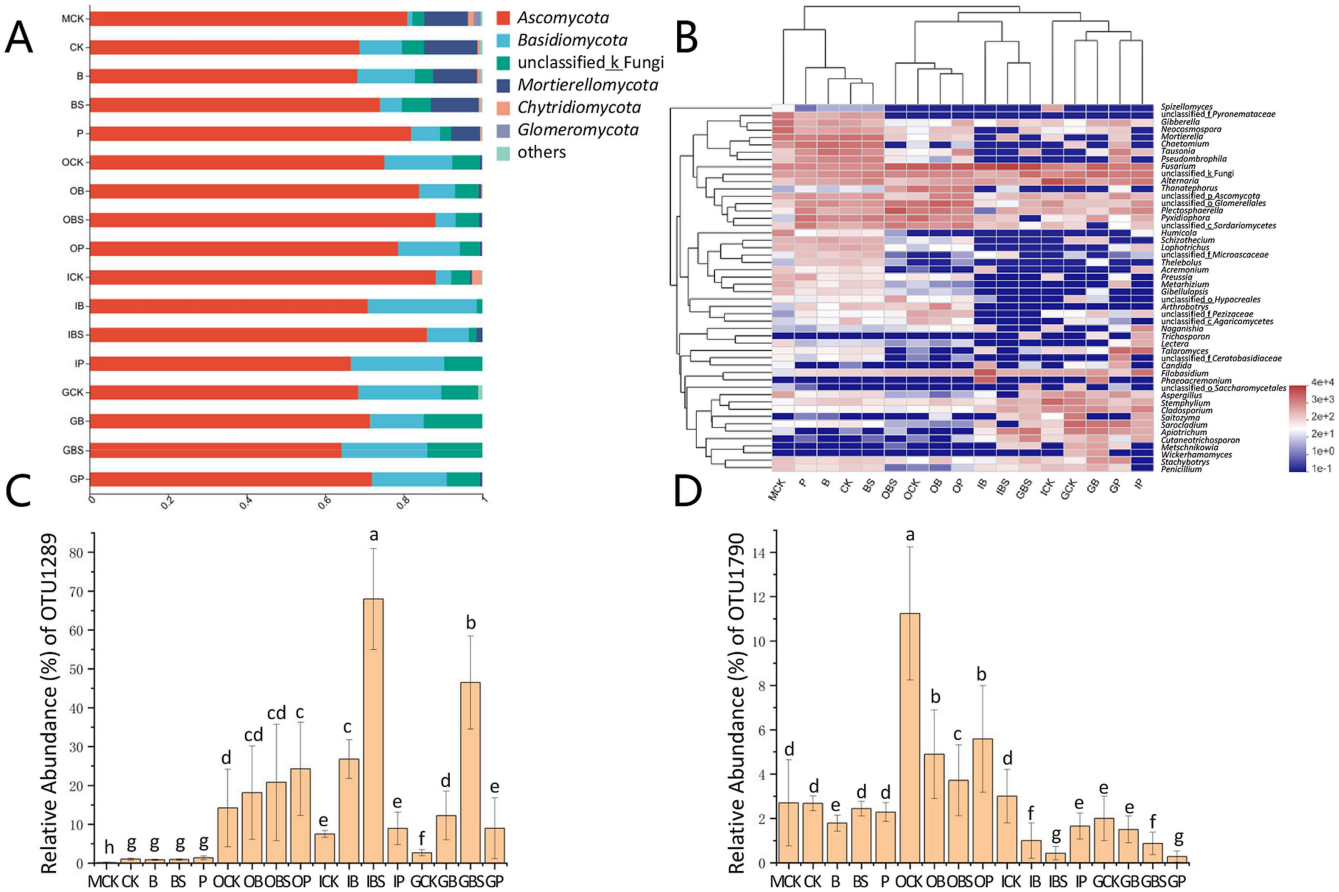
The fungal community composition of garlic tissues and rhizosphere. Composition of different fungal communities at the phylum **(A)** and genus **(B)** level. The relative abundance of different fungal phylum or genus within the different communities is shown in different colors. Others: the merging of taxa with the relative abundance less than 0.01. The difference in the relative abundance of OTU1289 **(C)** and OTU1790 **(D)** based on Kruskal-Wallis H test (*p* < 0.05). Bars with different letters are significantly different (*p* < 0.05). CK (irrigation water), B (*B. velezensis* TB918), BS (*B. velezensis* TB918 and sucrose) and P (commercial biocontrol agent *P. polymyxa*) depict rhizosphere soil samples and different treatments. M (margin soil), G (garlic cloves), I (inner sheaths), and O (outer sheaths) depict different sampling locations.

At the phylum level (Figure 4A; Supplementary Figure S3), the top two phyla, Ascomycota and *Basidiomycota* held the absolute dominant position, accounting for more than 80% of the entire fungal community. Notably, the relative abundance of *Mortierellomycota* reached 7.36–13.58% in the rhizosphere and margin soils, and its abundance was less than 1% in garlic tissues.

The phylum *Ascomycota* was the most diverse phylum, with *Alternaria*, *Plectosphaerella* and *Fusarium*, representing the majority genera. The relative abundances of *Ascomycota* were 64.13–88.12% in all the samples with minor differences, but the abundances of *Fusarium* significantly differed among the different treatments and niches (Figure 4B). For the suspected pathogenic fungus, in the garlic soil samples, the relative abundance of *Fusarium* was high in the field margin (MCK: 6.30%), while it was lower in treated rhizosphere (B:4.53%, BS:4.84%). In the outer sheath group, there was almost no difference in fungal community composition among treatments, and the relative abundances of *Fusarium* were similar between treatments. However, in the garlic clove (G) and inner sheath (I) groups, the relative abundance of *Fusarium* was higher in the BS treatment.

To explore this issue, after further annotation of species, the relative abundances of *F. concentricum* (OTU1289) and *F. proliferatum* (OTU1790) accounted for 96.5% of the *Fusarium* abundance (Supplementary Table S2). As shown in Figures 4C,D, the abundance of *F. concentricum* was relatively low in the rhizosphere and margin soils, but this species tended to aggregate on the outer sheaths, with no significant difference among the four treatments (OCK, OB, OBS, and OP). However, its abundance was only 7.69% (ICK) and 2.70% (GCK) in the control groups, but was significantly higher in the *Bacillus*-treated inner sheaths (IB: 26.77%) and cloves (GB: 12.25%), especially after the addition of sucrose (IBS: 68.01%, GBS: 46.47%). The relative abundance of *F. proliferatum*, which is the main causal agent of GDR, was similar to that of *F. concentricum* in the control groups of rhizosphere and garlic tissues (CK, OCK, ICK, and GCK). The application of biocontrol agents (*Bacillus* or *Paenibacillus*) was effective at reducing the relative abundance of *F. proliferatum* in different tissues. With the addition of sucrose, the relative abundances of *F. proliferatum* were even lower (OBS: 3.72%, IBS: 0.43%).

To further confirm the local pathogenic fungus causing GDR, 4 fungal strains from infected garlic bulbs were isolated. Internal transcribed spacer (ITS) region and RNA polymerase II second largest subunit (RPB2) sequences of the isolated fungi were phylogenetically analyzed to determine their taxonomic identity (Supplementary Figure S4). BLAST analysis of the ITS sequences showed that *F. solani* GCF1, *F. equiseti* GCF2, *F. solani* GCF3, and *F. proliferatum* GCF4 had 89.1, 93.2, 86.6, and 99.6% sequence similarity with OTU678, OTU1753, OTU1587, and OTU1790, respectively. Whereas, the OTUs of GCF1-3 were low-abundance OTUs, we also selected all the isolated strains to test their ability to cause GDR. Pathogenicity assays revealed that the tested isolates caused symptoms on tested cloves (Supplementary Figure S5), like naturally infected garlic. The pathogen was reisolated and confirmed to be *F. proliferatum* according to ITS sequence identification and morphological characteristics. In addition, replacing glucose with sucrose in PDA did not affect the growth and colony morphology of 4 *Fusarium* (Supplementary Figure S6).

### *B. velezensis* TB918 enhanced the stability of microbial community structure on garlic

**Figure 5:**
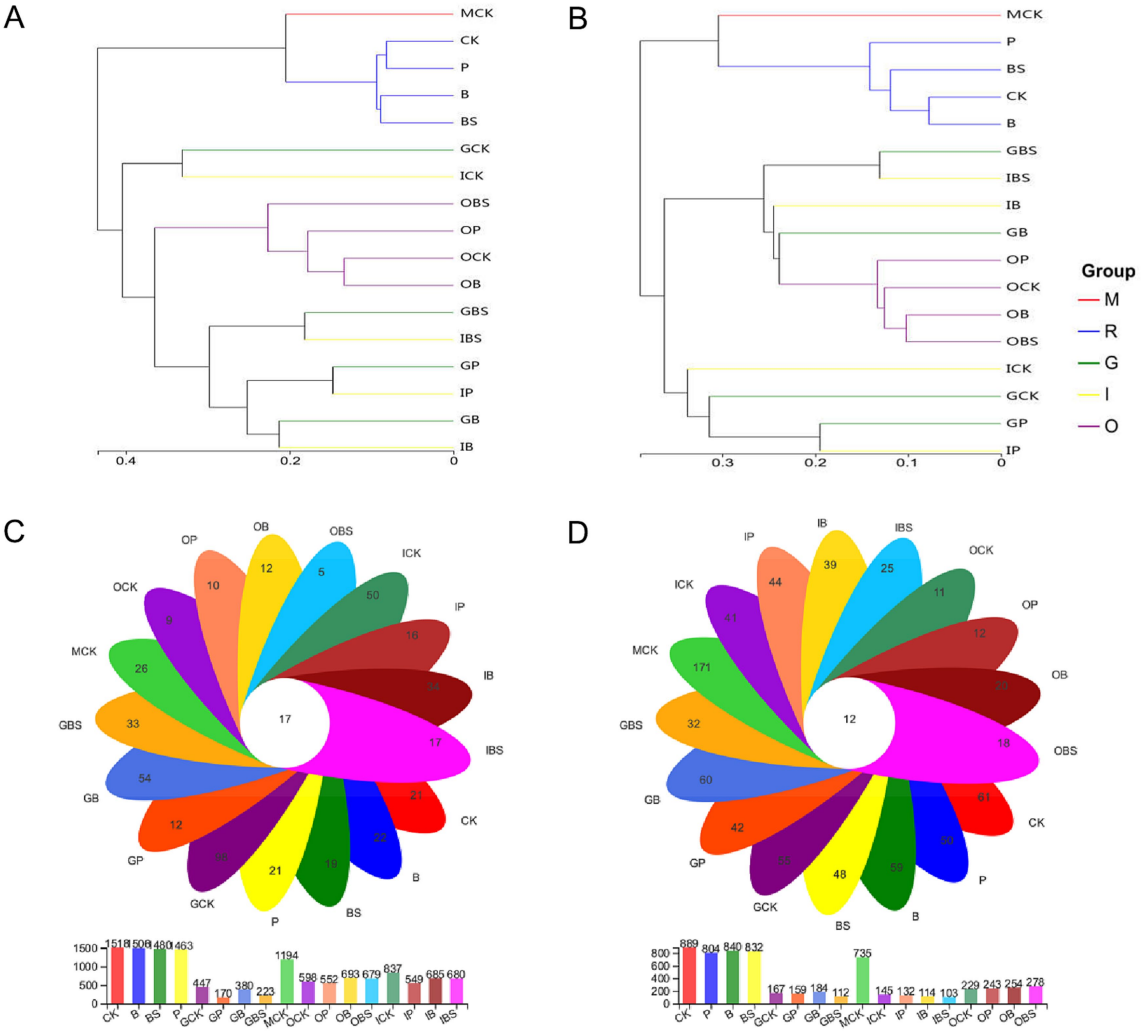
Microbial clustering and distribution of garlic tissues and rhizosphere. UPGMA-Tree Clustering analysis on genus level of soil bacterial **(A)** and fungal **(B)** community based on the Unweighted Pair group Method with Arithmetic Mean (UPGMA) in garlic with different treatments. Samples were clustered into five groups: M: margin soil group, R: rhizosphere soil group, G: garlic cloves group, I: inner sheaths group, O: outer sheaths group. The Venn diagram of bacterial **(C)** and fungal **(D)** OTUs of garlic tissues and rhizosphere. CK (irrigation water), B (*B. velezensis* TB918), BS (*B. velezensis* TB918 and sucrose) and P (commercial biocontrol agent *P. polymyxa*) represent rhizosphere soil samples and different treatments. M (margin soil), G (garlic cloves), I (inner sheaths) and O (outer sheaths) represent different sampling locations. Flower plot showing numbers of specific OTUs found in each treatment (in the petals), and common OTUs for different treatments (in the center).

Each branch on the UPGMA phylogenetic tree represented one sample of garlic tissue or soil microbiota (Figures 5A,B). Specifically, the clusters clearly differed according to the sampling area (rhizosphere or margin soil and outer sheath) for both bacteria and fungi. The microbial communities from rhizosphere and outer sheaths clustered independent of treatments, while that from inner sheaths and garlic cloves of the same treatments clustered in both bacteria and fungi.

The Venn diagram visually displays the shared and specific occurrence of OTUs in different compartments (Figures 5C,D). Our analysis of the OTUs revealed that the total number of bacteria or fungi in the margin (MCK) and rhizosphere (R) soil was more than that in the outer (O), inner sheath (I) or garlic clove (G), as well as the number of OTUs in R was higher than that in MCK. The total number of bacterial OTUs in the G group and the number of fungal OTUs in the G/I group were lower than those in the other groups. Interestingly, though the total number of bacteria was low, there were more specific bacterial OTUs in all garlic clove (G) treatments, which might be related to the fact that many of them were endophytic bacteria in plants. For fungi, more specific OTUs were found in the margin (MCK) and rhizosphere (R) soils than in the other sampling areas, while the outer sheath (O) had the least number of specific OTUs, although TB918 (B) and sucrose (BS) could increase the number of specific OTUs. In other words, cultivation practices could alter the fungal structure in the original soil and reduce its richness.

**Figure 6:**
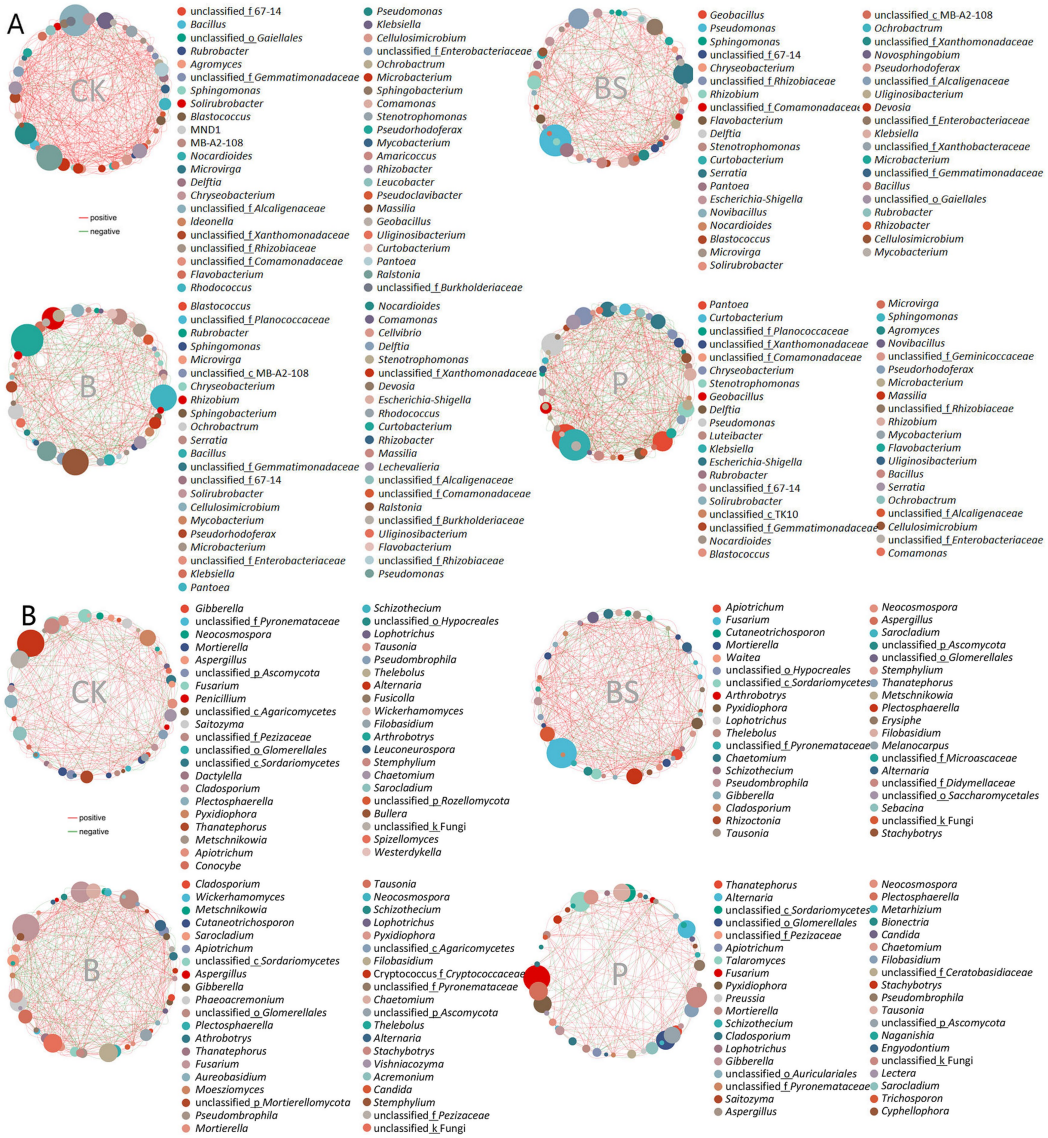
The co-occurrence network diagram of garlic rhizosphere inoculating biocontrol agents based on Spearman’s correlation analysis of the abundances for the 50 most abundant microbial genera. **(A)** Network diagram based on the bacterial genus level. **(B)** Network diagram based on fungal genus level. The red line indicates a positive correlation and the green line indicates a negative correlation. The size of the dots represents the average abundance of the genus. CK (irrigation water), B (*B. velezensis* TB918), BS (*B. velezensis* TB918 and sucrose), and P (commercial biocontrol agent *P. polymyxa*) represent rhizosphere soil samples with different treatments.

To explore the comprehensive microbial community dynamics and reveal the co-occurrence patterns at the phylogenetic level, network analysis was performed based on the 50 top abundant microbial genera in soils and garlic tissues. The appearance of TB918 caused B (295) and BS (304) to have fewer edges than did CK (389) or *P* (401) in the bacterial networks, while in contrast to fungi, B (376) and BS (326) had more edges than did CK (271) and *P* (182) in the networks (Figure 6; Supplementary Table S5). The trends of average network transitivity were basically consistent with those of the network edge for both bacteria and fungi. On average, the addition of exogenous microorganisms or its combination with sugar reduced the average path length (1.86–2.04) in the bacterial network (CK: 2.17), but only the application of TB918 with or without sucrose decreased the average path length (2.06–2.09) in the fungal network (CK: 2.34). The average negative correlations in microbial co-occurrence networks were evaluated (Supplementary Table S5). After the addition of TB918 only, the bacterial and fungal networks comprised a relatively large proportion of negative correlations compared with those in the CK or other treatments.

### *Bacillus* facilitates the growth and colonization of *Pseudomonas* by forming consortia

To further validate the positive effect of *B. velezensis* TB918 on *Pseudomonas*, three *Pseudomonas* strains were isolated from tissues and rhizosphere of the TB918 and sucrose inoculated garlic, named strains GP1, GP2, and GP3. After phylogenetic analysis based on the 16S rRNA and *gyrB* genes sequences amplified from the extracted bacterial DNA of each *Pseudomonas* isolate, the species names of three isolates were identified (Supplementary Figure S4). *P. brenneri* GP1, *P. fluorescens* GP2, and *P. gessardii* GP3 showed 99.74, 99.73, and 97.71% sequence similarity with OTU239, OTU1794, and OTU1062, respectively.

**Figure 7:**
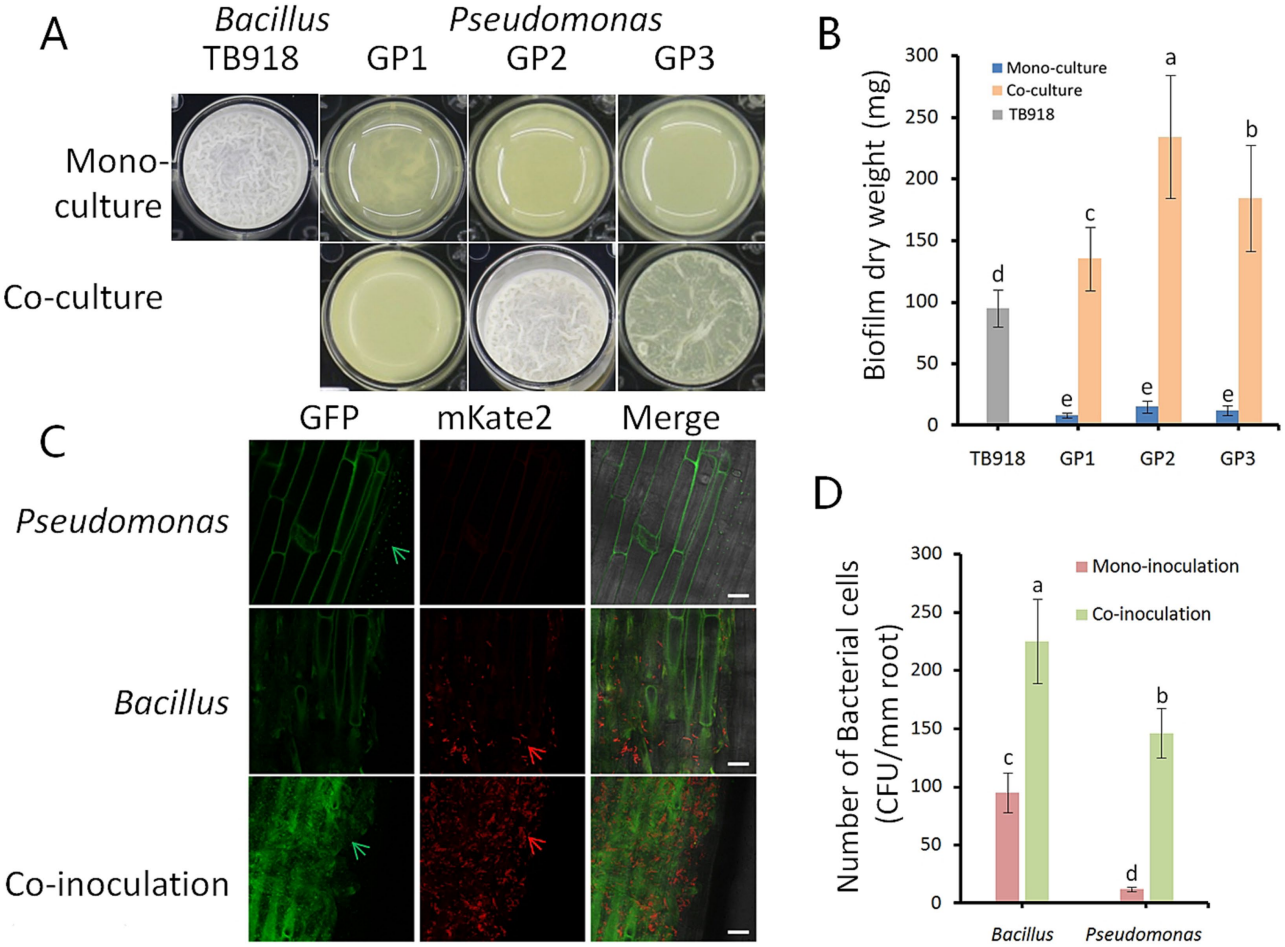
Influence of *Bacillus* on biofilm phenotype and colonization of isolated *Pseudomonas*. **(A)** Biofilm phenotype of the isolated *Pseudomonas* strains. GP1-3 represents different Pseudomonas strains. Well diameter is 16.5 mm. **(B)** Pellicle biomass quantified by dry weight. Pellicles were cultivated in MSgg medium for 24 h. TB918 represents *B. velezensis* TB918, GP1-3 represent different isolates. Gray and blue bars represent mono-culture of TB918 and GP1-3, respectively. Co-culture means mixed cultivation of TB918 and GP1-3 either. The bars represent the standard error. The letters above the columns indicate statistically significant differences based on the index using the Student’s *t*-test (*p* < 0.05). **(C)** The roots of 14-day-old garlic were colonized by *B. subtilis* 3610 (mKate2, red arrows), *P. fluorescens* GP2 (GFP, green arrows), and both. LSCM pictures are representative of at least 10 independent garlic roots. Scale bar represents 20 μm. **(D)** The difference in the garlic root colonization between *Bacillus* and *Pseudomonas* in mono- or co-inoculation condition was determined by counting colony forming unit (CFU) per mm root length. The bars represent the standard error. The letters above the columns indicate statistically significant differences based on the index using the Student’s *t*-test (*p* < 0.05).

Whether three *Pseudomonas* isolates act synergistically with *B. velezensis* TB918 for biofilm formation was investigated in an *in vitro* coculture assay with MSgg medium (Branda et al., 2001). Measurements of biofilm biomass in the coculture assays showed that all three *Pseudomonas* isolates had enhanced biofilm formation (Figures 7A,B). These isolates produced almost no biofilm biomass under monoculture conditions, but the coculture biofilm dry weight of GP1, GP2, and GP3 increased 1.2–2.5-fold compared to the TB918 monoculture pellicle. In order to test the effect of sucrose on bacterial growth, *Pseudomonas* isolates and *Bacillus* strains were cultured on the LB solid medium (1.5% agar) with 0.5% (w/v) sucrose. It was found that sucrose can promote the solid surface motility of *Bacillus*, but this phenomenon was not observed in *Pseudomonas* culture (Supplementary Figure S6). Otherwise, the tablet confrontation test (Supplementary Figure S7) showed that the growth of four *Fusarium* isolates was apparently inhibited by TB918 and GP2. Whereas GP1 and GP3 had almost no antagonistic effect on *Fusarium* isolates.

The roots were observed 48 h after inoculation with strain 3610 expressing mKate2 and strain GP2 expressing GFP constitutively. Images from laser scanning confocal microscope (LSCM) suggested *P. fluorescens* GP2 colonized the roots of garlic at substantially lower abundance than *B. subtilis* 3610 under hydroponic conditions (Figure 7C). However, these two species were able to co-colonize the garlic roots. Root recovery counting revealed 12.2- and 2.4-fold increase in the colonization efficiencies for GP2 and 3610, respectively, in co-inoculation compared to mono- inoculation (Figure 7D).

## Discussion

Dugan et al. (2019) noted that up to 77% of seemingly healthy bulbs exhibited GDR symptoms after being stored for 9–16 months at harvest, indicating that garlic cloves used as seeds, even if they appeared healthy, could still be infected by *Fusarium*. Thus, sowing healthy cloves and protecting the plantlet during the early growth stages are crucial for reducing the incidence of GDR. To explore an efficient and safe method for controlling GDR, the *B. velezensis* TB918 strain, sucrose, and a commercial bioagent were applied via root irrigation, and the changes in the structure and diversity of the microbial community of garlic plants grown in the field after application were investigated. In this study, the field experiment results confirmed that the biocontrol agent *B. velezensis* TB918 and sucrose had outstanding effects on GDR control.

Further microbiome analysis revealed that the alpha indices in the treatment groups were not significantly different from those in the control group. This analysis indicated that the content and stability of the indigenous microbiota were high (Yao et al., 2021) and that the introduction of *B. velezensis* TB918 and sucrose did not change the richness or diversity of the microbial community in the rhizosphere or tissues of garlic. On the other hand, the relative abundance analysis of *Bacillus*, *Pseudomonas* and *Fusarium* implied that plant-beneficial and plant-harmful microorganisms were influenced by the addition of TB918 and sugar. The data implied that the exogenous addition of *B. velezensis* TB918 and sucrose could effectively synergistically increase the content of beneficial rhizosphere bacteria such as *Pseudomonas* and significantly inhibit the harmful genus *Fusarium*, which could enhance the structural stability and diversity of the garlic microbial community and improve the soil microecosystem for garlic cultivation in farmland. These results were consistent with our previous study that sucrose could promote root colonization and the disease control efficacy against some soil-borne phytopathogens by *B. subtilis* via “a levan detour” and shape the rhizomicrobiome by increasing the abundance of beneficial bacteria such as *Pseudomonas* (Tian et al., 2021). In this study, the results further validated and expanded the hypothesis that sucrose could promote the colonization of *Bacillus* on plants.

Three *Pseudomonas* strains were specifically isolated to validate the positive influence of *B. velezensis* TB918 on *Pseudomonas* biofilm formation. The result was consistent with the amplicon data, since TB918 treatments could increase the relative abundance of 3 corresponding OTUs, OTU239, OTU1794, and OTU1062. Because OTU1794 had the highest abundance among the OTUs of *Pseudomonas* and showed the most enhanced biofilm phenotype with *B. velezensis* TB918, we chose strain GP2 as a representative for co-colonization assay with *Bacillus*. Given the strong influence of *B. velezensis* TB918 on the biofilm formation of *Pseudomonas*, we reasoned that *Bacillus* could stimulate the root colonization of *Pseudomonas* synergistically in garlic. The microbiome analysis and performance of two species consortia in the root also indicated that *Bacillus* can induce the enrichment and colonization of *Pseudomonas* in the garlic rhizosphere and bulb. These results are consistent with previous reports that *B. velezensis* SQR9 facilitates the growth and biofilm formation of *P. stutzeri* XL272 isolated from cucumber rhizosphere by metabolic cross-feeding and chemotactic attraction (Sun et al., 2022). *Bacillaceae* species were also reported to promote the colonization of rhizobia (Han et al., 2020) and stimulate indigenous soil *Pseudomonas* populations that enhance plant disease suppression (Tao et al., 2020). *B. subtilis* 3610 strain was often chosen as the model system to study bacterial movement on solid surfaces and the colonization in roots (Kearns and Richard, 2010; Tian et al., 2021). Otherwise, *B. velezensis* is a species closely related to *B. subtilis* (Xu et al., 2013). Given that *B. velezensis* TB918 could not be genetically manipulated, we believed that strain 3610 in the co-colonization on garlic roots with GP2 was representative of strain TB918. Indeed, our results herein need to be confirmed by extended studies when *B. velezensis* TB918 could be genetically manipulated or specifically detected.

The antagonistic activities of strain TB918 and GP2 against four *Fusarium* isolates were confirmed by tablet confrontation test. It is worth noting that recent research has demonstrated that *Pseudomonas* species can also be effective against soil-borne fungal pathogens in non-plant systems. For example, Yu et al. (2025) reported that *Pseudomonas chlororaphis* significantly alleviated a soil-borne disease in the mycosphere of *Morchella importuna*, a cultivated edible mushroom, by suppressing *Paecilomyces penicillatus*. This study highlights the versatility of *Pseudomonas* in disease control beyond green plants and suggests that its biocontrol potential could extend to broader soil environments, including fungal cropping systems. *Pseudomonas* can be beneficial not only in plant disease suppression but also in fungal cultivation systems.

As reported, *F. proliferatum* has been signaled as the main causal agent of GDR and has a worldwide wide host range spanning different climate zones (Mondani et al., 2021). Our results also indicated that *F. proliferatum* was the main pathogenic fungus causing local GDR. Though *F. concentricum* also had a high abundance among the OTUs of *Fusarium* and has been reported to have plant pathogenicity, but it could also be an endophytic fungus with a biocontrol function for promoting growth and insecticidal activity (Zhang et al., 2022). Similar to that of certain *Pseudomonas* species, the relative abundance of *F. concentricum* was also elevated with *Bacillus* inoculation and sucrose supplementation. *F. concentricum* is considered as the pathogen for fruit rot of banana and tangerine (Abd Murad et al., 2017; Xiao et al., 2022) in field, storage and postharvest procedures, which contributes to yield loss and reduced quality to varying degrees. Zhang et al. (2022) reported that the endophytic fungus *F. concentricum,* which was isolated from the medicinal plant *Anoectochilus roxburghii*, could produce tryptophan derivatives and pyridone alkaloids, and these compounds displayed a range of biological activities, including antibacterial, antifungal and insecticidal properties (Chang et al., 2022). The results of fungal communities’ composition corresponded to the incidence rate and disease severity of GDR, indicating that the application of sugar could effectively enhance the colonization and competitive abilities of *Bacillus* by garlic. Plant–microbe interactions are commonly context dependent because the microbes associated intimately with plants change their infection modes dynamically according to host conditions and the environment (Cheng et al., 2019; Drew et al., 2021). In other words, even within the same host plant, a microorganism can switch between pathogenic and beneficial types without genomic changes, depending only on the environmental conditions (Hiruma et al., 2023). No report has proven that *F. concentricum* can cause dry rot in garlic. These results suggested that supplementation with *Bacillus* and sucrose might inhibit pathogenic species, resulting in an increase in nonpathogenic species of *Fusarium*.

The results of hierarchical clustering and principal coordinate analysis showed that the microbial composition varied not only between garlic tissues and rhizosphere soil but also among diverse tissues, indicating spatial differences in the composition of garlic microbiota. The microbial community of the outer sheath was different from that of both the rhizosphere soil and the internal tissues of garlic, implying that the outer sheath had a unique ecological status. The structures of the microbial communities in garlic cloves and inner sheath were similar, and that the responses to the addition of exogenous microorganisms were consistent. The total number of OTUs in the G/I group were lower than those in the other groups, indicating the role of multiple layers of the sheath in hindering microbial entry, especially for fungi. The inhibitory effect of garlic sheaths on the distribution of microbiota suggested that they may play an important role in disease defense. As a non-endophytic beneficial bacterium, TB918 had little impact on the diversity and composition of the microbiota in garlic cloves and inner sheaths.

Network-based analytical methods have proven useful for studying systems with complex interactions (Matchado et al., 2021). The data of network analysis indicated that the bacterial community network exhibited a significant increase in size and complexity following the introduction of TB918 and sugar in garlic cultivation, whereas the fungal community displayed a contrasting trend. To estimate the microbiome stability of garlic after inoculation with exogenous bacteria, the average negative correlations in microbial co-occurrence networks were evaluated. It has been reported that the existence of negative interactions is conducive to co-oscillation stability in microbial communities and promotes the stability of networks (Li et al., 2023). The result implied the role of TB918 in enhancing the stability of microbial community structure on garlic.

Starting with the exogenous application of the bioinoculant *B. velezensis* TB918 and low-cost additive sugar, our work indicated that not only could TB918 effectively control GDR but also that sugar could notably enhance the colonization and competitive abilities of *Bacillus* in garlic. Furthermore, the network structure of bacteria and fungi in the rhizosphere and tissues of garlic plants was significantly affected by TB918 and sugar. Paired with sucrose, the biocontrol agent TB918 could regulate the structure of *in situ* rhizosphere microorganisms, inhibit pathogenic microorganisms, mobilize beneficial microorganisms within the environment-dominated variable microbiota and promote the transition of microbes from passive service to active service in the rhizosphere. By implementing reasonable soil management measures, we can regulate the structure and function of rhizosphere variable microbial communities to support the green development of plant disease prevention and control.

## Conclusion

*B. velezensis* TB918 and sucrose strongly prevented and controlled GDR during storage. The microbial communities of the rhizosphere and different garlic bulb compartments were investigated in this research. In particular, although the introduction of *B. velezensis* TB918 and sucrose hardly changed the richness and diversity of the microbial community in the rhizosphere and tissues of garlic, the relative abundance analysis of *Bacillus*, *Pseudomonas* and *Fusarium* implied that plant-beneficial and plant-harmful microorganisms were influenced. Furthermore, TB918 and sucrose could synergistically increase the content of beneficial rhizosphere bacteria such as *Pseudomonas*, significantly inhibit the harmful species of *Fusarium*, enhance the structural stability of the garlic microbial community and improve the soil microecosystem for garlic cultivation in farmlands.

## Data Availability

The raw sequence data reported in this paper have been deposited in the Genome Sequence Archive (Chen et al., 2021) in National Genomics Data Center (CNCB-NGDC 2022), China National Center for Bioinformation/Beijing Institute of Genomics, Chinese Academy of Sciences that are publicly accessible at https://ngdc.cncb.ac.cn/gsa. The BioProject numbers is PRJCA026428, including the 16S rRNA and *gyrB* gene sequencing data (SAMC4667137-SAMC4667139), ITS rRNA and RPB2 gene sequencing data (SAMC4581208-SAMC4581211) and metagenomic data (SAMC4667140-SAMC4667143).

## Footnotes

^1^ www.majorbio.com

^2^ http://www.mothur.org/wiki/Schloss_SOP#Alpha_diversity

^3^ http://drive5.com/uparse/

^4^ http://qiime.org/

## Supplementary material

Supplementary Figure S1 Relative rank-abundance curves for the OTUs of bacteria **(A)** and fungi **(B)** in the garlic tissues and rhizosphere soil samples. The OTUs reads of different samples are shown in different colors. CK (irrigation water), B (*B. velezensis* TB918), BS (*B. velezensis* TB918 and sucrose), and P (commercial biocontrol agent *P. polymyxa*) depict rhizosphere soil samples and different treatments. M (margin soil), G (garlic cloves), I (inner sheaths), and O (outer sheaths) depict different sampling locations.

Supplementary Figure S2 Rarefaction curves of OTUs for the garlic tissues and rhizosphere soil samples. Shannon curves for bacteria **(A)** and fungi **(B)**. Chao1 curves for bacteria **(C)** and fungi **(D)**.

Supplementary Figure S3 Bubble plots demonstrated the relative abundances of abundant phyla based on 16S rRNA **(A)** and ITS **(B)** amplicon sequencing. Others: the merging of taxa with the relative abundance less than 0.01.

Supplementary Figure S4 Phylogenetic tree reconstructed of the isolated strains with Mega 7.0 software using neighbor-joining method. **(A)** Based on the 16S rRNA and *gyrB* sequences of *Pseudomonas.* **(B)** Based on ITS and RPB2 sequences of *Fusarium*.

Supplementary Figure S5 Pathogenicity test performed on garlic cloves. Control: inoculated with sterile distilled water. GCF1-4: *F. solani* GCF1, *F. equiseti* GCF2, *F. solani* GCF3, and *F. proliferatum* GCF4.

Supplementary Figure S6 The growth of *Fusarium* and *Pseudomonas* strains in media supplemented with and without sucrose. LBS was LB media with 0.5% (w/v) sucrose. PSA was prepared from PDA media, replacing glucose with 2% (w/v) sucrose. 3610: *B. subtilis* 3610, TB918: *B. velezensis* TB918, GP1-3: *P. brenneri* GP1, *P. fluorescens* GP2 and *P. gessardii* GP3, GCF1-4: *F. solani* GCF1, *F. equiseti* GCF2, *F. solani* GCF3, and *F. proliferatum* GCF4.

Supplementary Figure S7 Tablet antagonism of *B. velezensis* TB918 and three *Pseudomonas* isolates against four *Fusarium* isolates.The schematic diagram means: the center of each tablet is *Fusarium* isolates, the four quadrants are in order as *P. fluorescens* GP2, *P. gessardii* GP3, *P. brenneri* GP1 and *B. velezensis* TB918. Four *Fusarium* isolates are: *F. solani* GCF1, *F. equiseti* GCF2, *F. solani* GCF3, and *F. proliferatum* GCF4.
