# Supplementary figures and images for: Bacillus velezensis TB918 mitigates garlic dry rot disease by forming consortia with Pseudomonas in the rhizosphere and bulb
Source: Front Microbiol. 2025 Apr 15;16:1567108. doi: 10.3389/fmicb.2025.1567108 (PMC12037484; doi:10.3389/fmicb.2025.1567108)

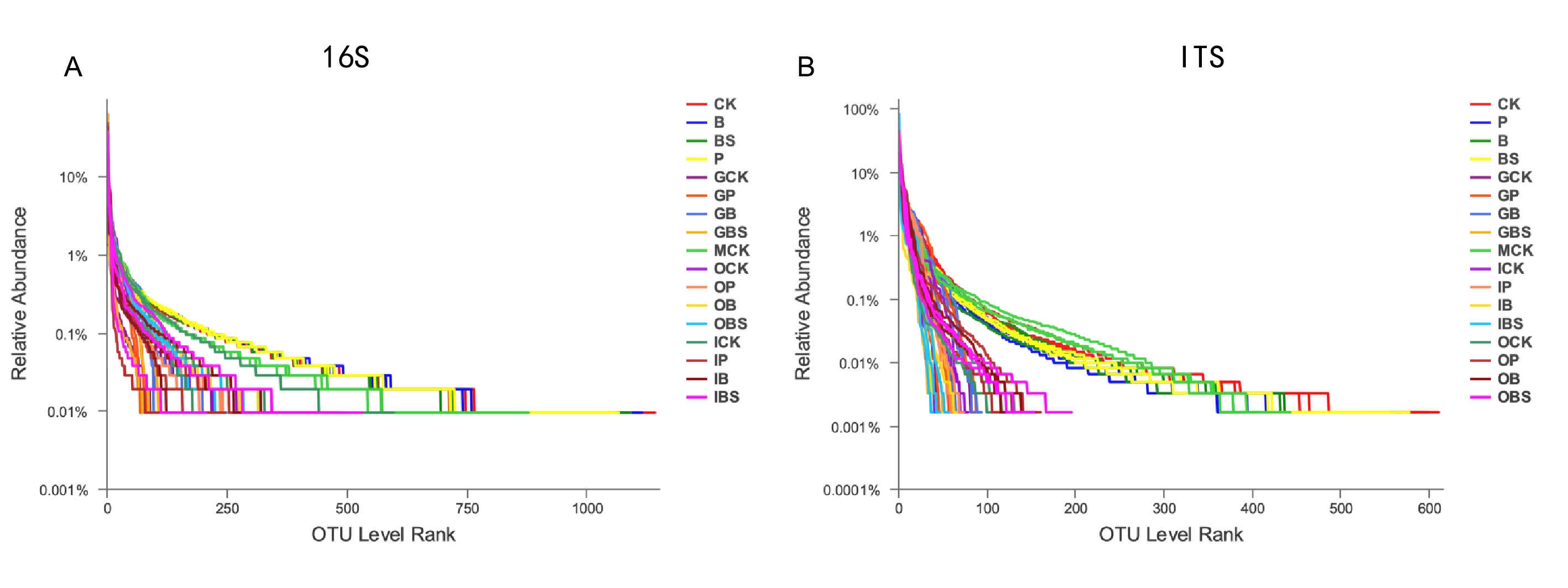

Supplement: Supplementary Figure S1 — Relative rank-abundance curves for the OTUs of bacteria (A) and fungi (B) in the garlic tissues and rhizosphere soil samples. The OTUs reads of different samples are shown in different colors. CK (irrigation water), B (B. velezensis TB918), BS (B. velezensis TB918 and sucrose), and P (commercial biocontrol agent P. polymyxa) depict rhizosphere soil samples and different treatments. M (margin soil), G (garlic cloves), I (inner sheaths), and O (outer sheaths) depict different sampling locations. [file Image_1.TIF]

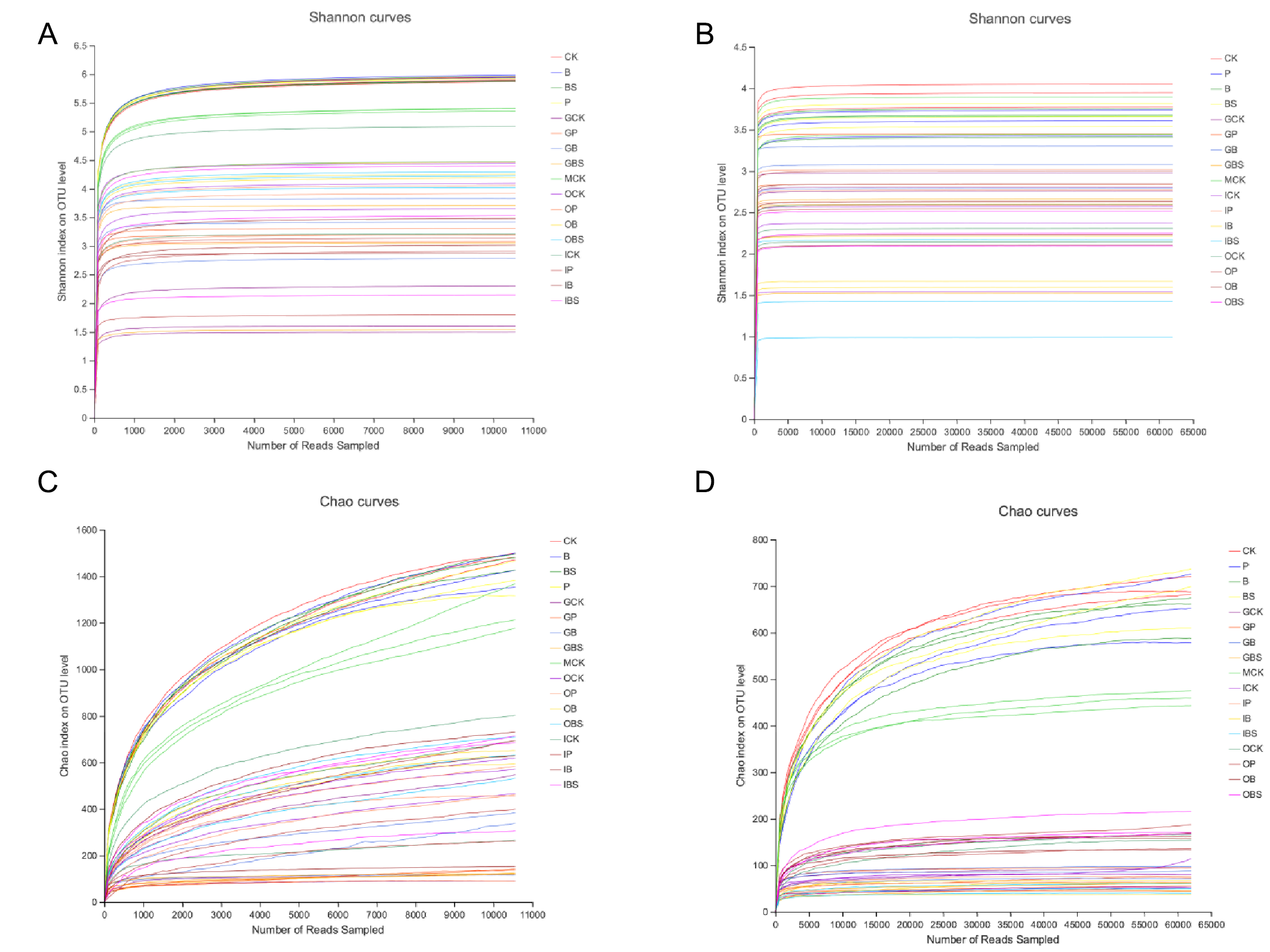

Supplement: Supplementary Figure S2 — Rarefaction curves of OTUs for the garlic tissues and rhizosphere soil samples. Shannon curves for bacteria (A) and fungi (B). Chao1 curves for bacteria (C) and fungi (D). [file Image_2.TIF]

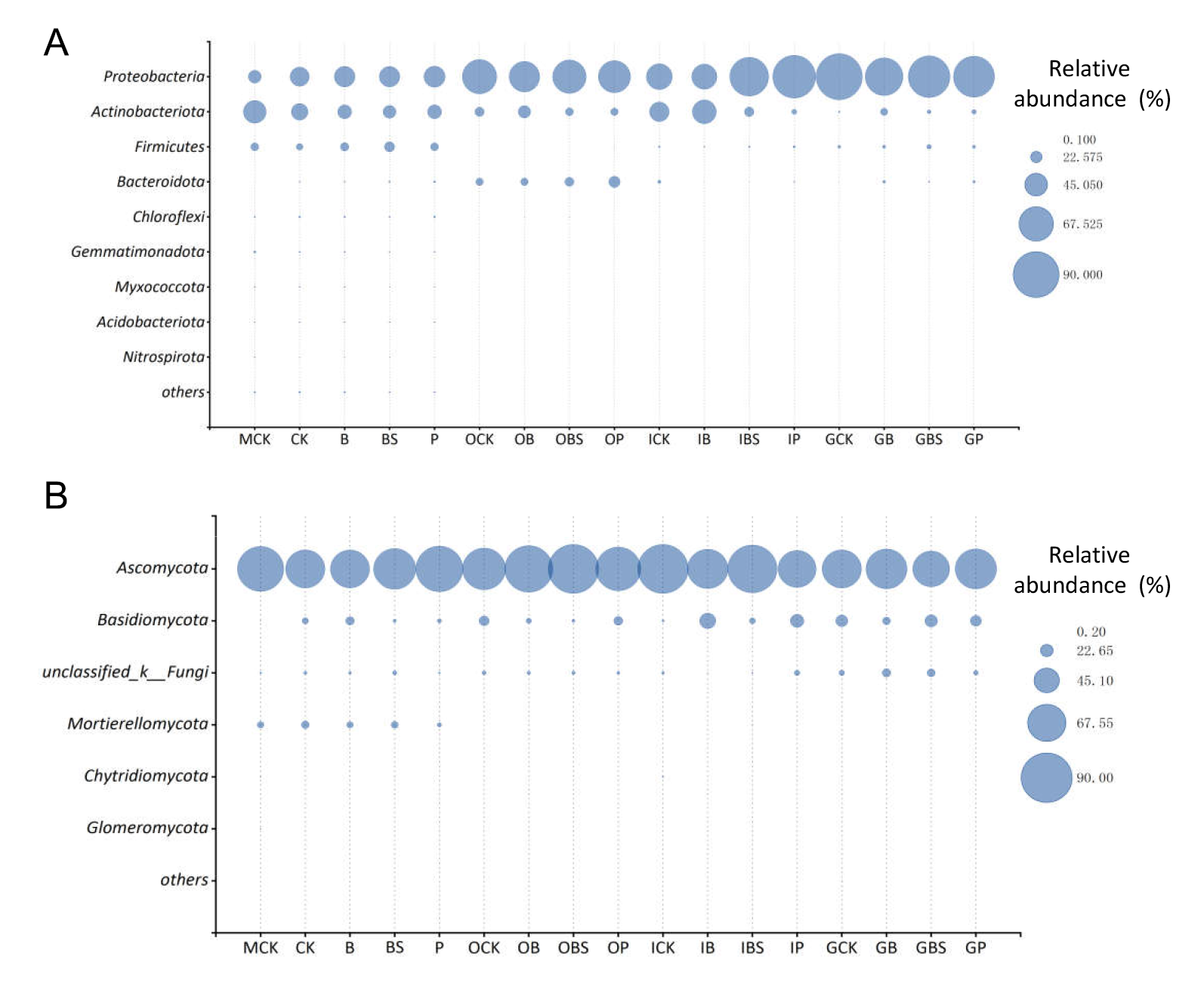

Supplement: Supplementary Figure S3 — Bubble plots demonstrated the relative abundances of abundant phyla based on 16S rRNA (A) and ITS (B) amplicon sequencing. Others: the merging of taxa with the relative abundance less than 0.01. [file Image_3.TIF]

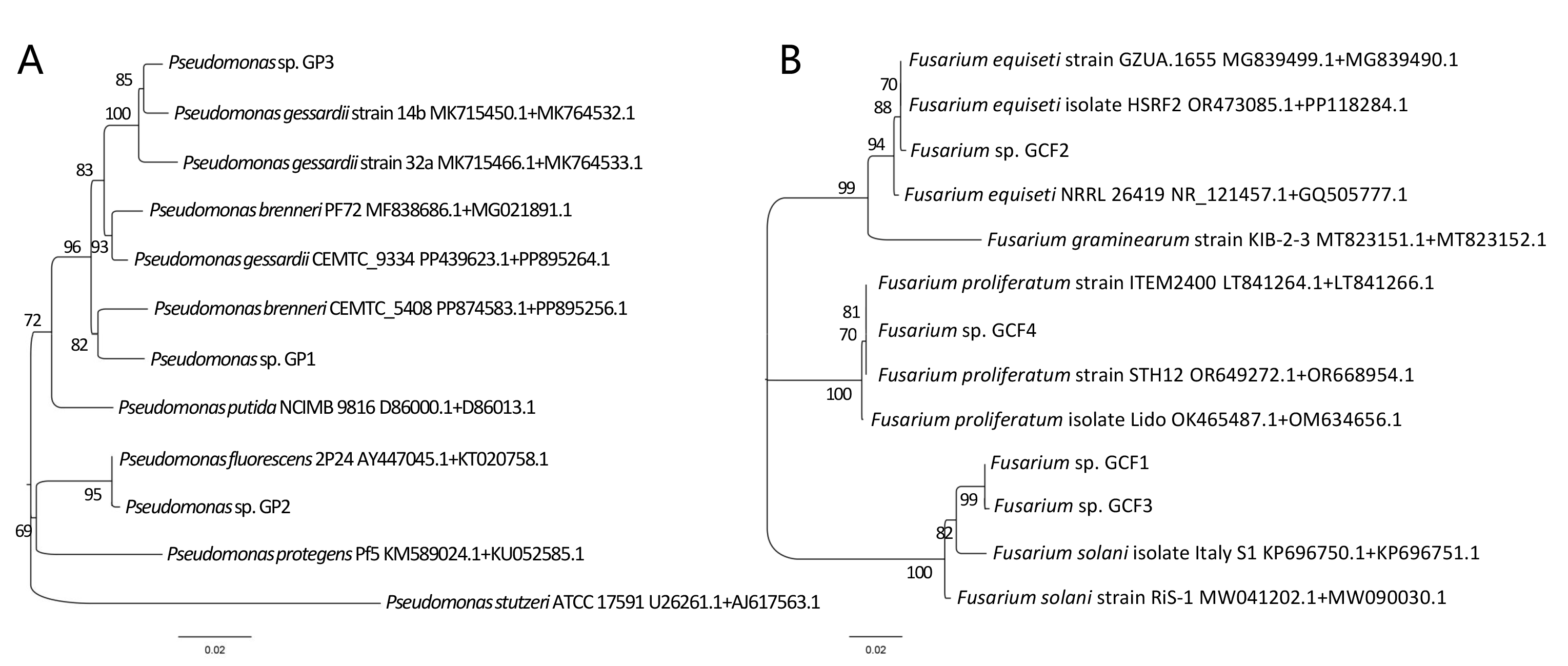

Supplement: Supplementary Figure S4 — Phylogenetic tree reconstructed of the isolated strains with Mega 7.0 software using neighbor-joining method. (A) Based on the 16S rRNA and gyrB sequences of Pseudomonas. (B) Based on ITS and RPB2 sequences of Fusarium. [file Image_4.TIF]

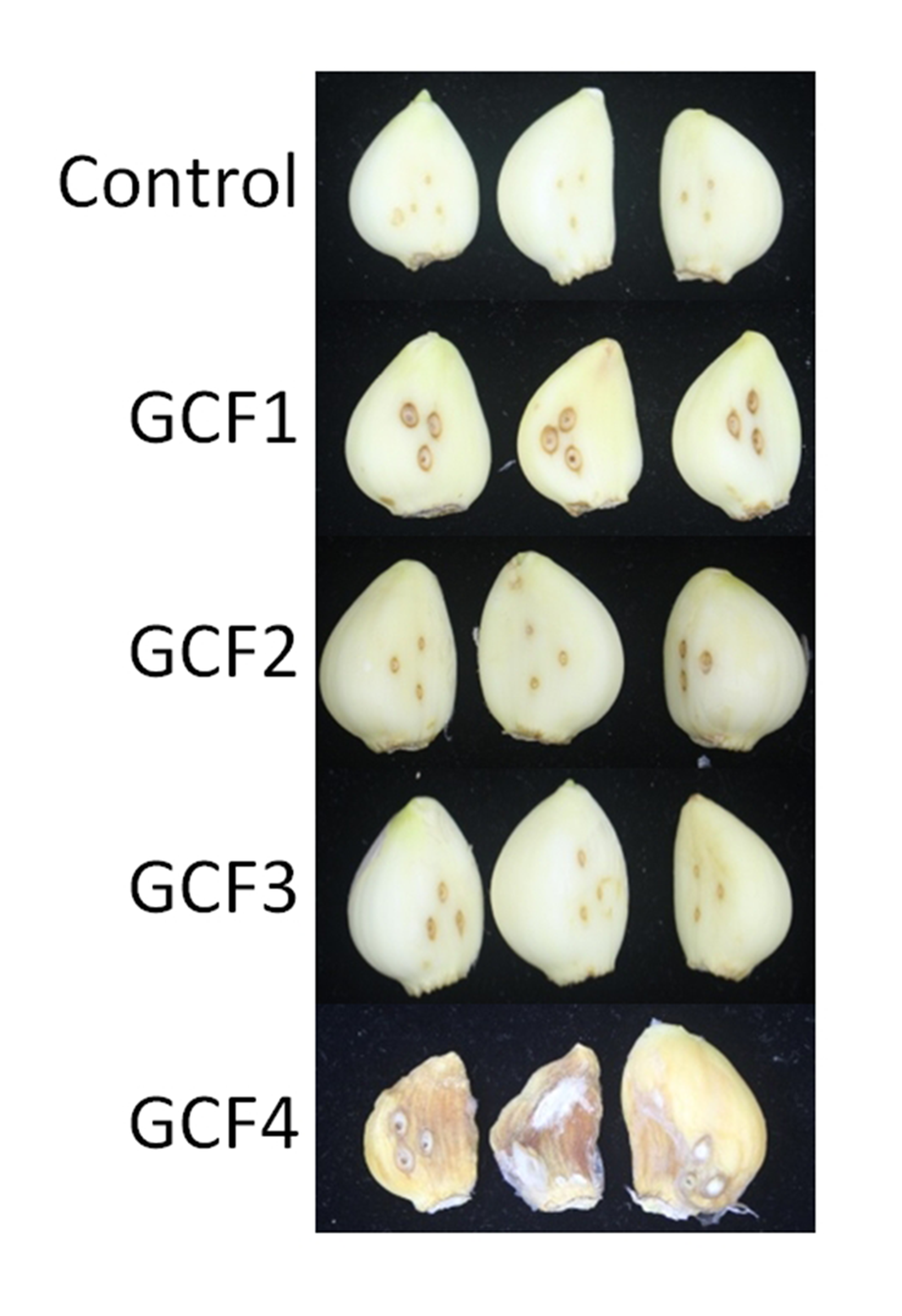

Supplement: Supplementary Figure S5 — Pathogenicity test performed on garlic cloves. Control: inoculated with sterile distilled water. GCF1-4: F. solani GCF1, F. equiseti GCF2, F. solani GCF3, and F. proliferatum GCF4. [file Image_5.TIF]

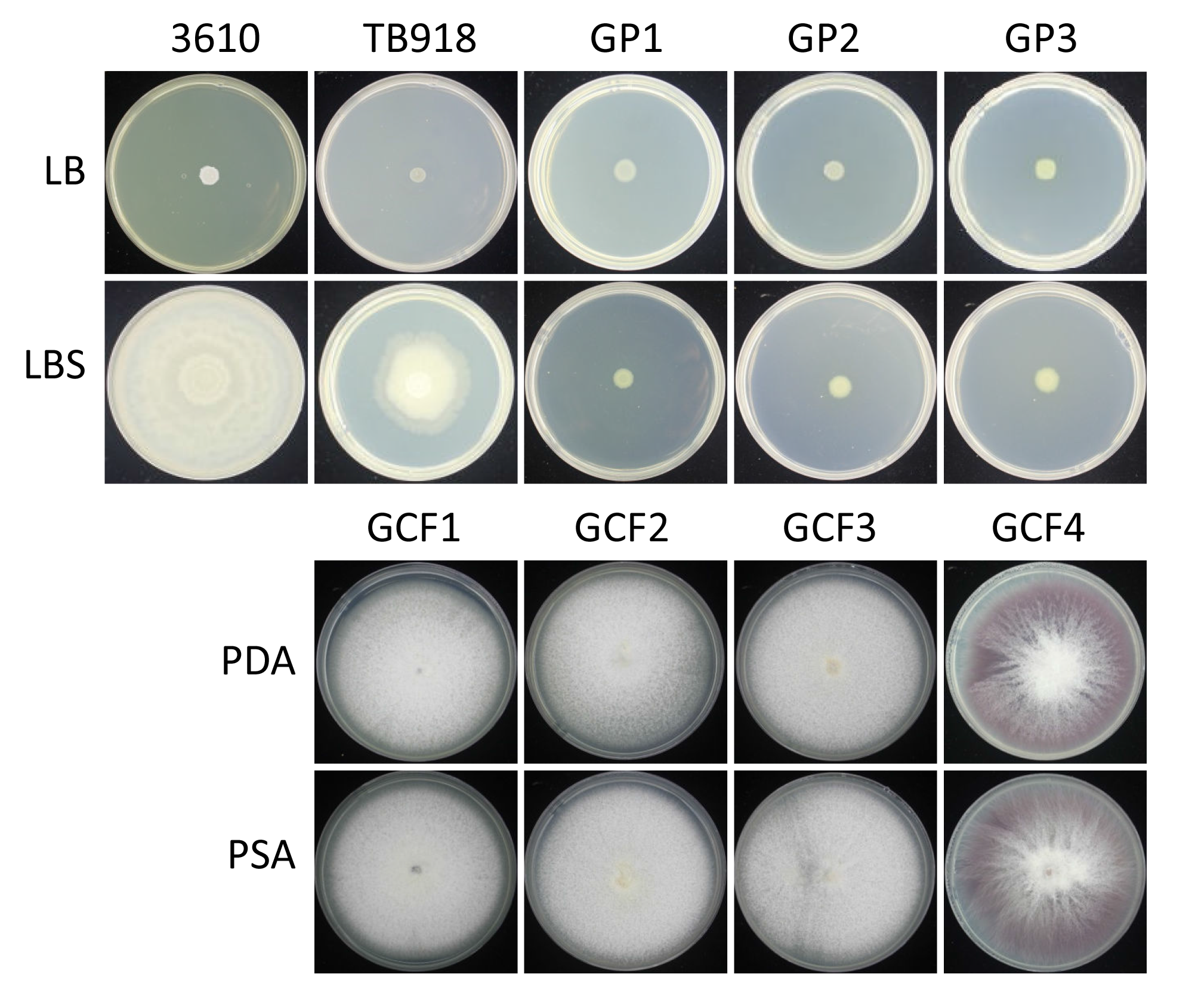

Supplement: Supplementary Figure S6 — The growth of Fusarium and Pseudomonas strains in media supplemented with and without sucrose. LBS was LB media with 0.5% (w/v) sucrose. PSA was prepared from PDA media, replacing glucose with 2% (w/v) sucrose. 3610: B. subtilis 3610, TB918: B. velezensis TB918, GP1-3: P. brenneri GP1, P. fluorescens GP2 and P. gessardii GP3, GCF1-4: F. solani GCF1, F. equiseti GCF2, F. solani GCF3, and F. proliferatum GCF4. [file Image_6.TIF]

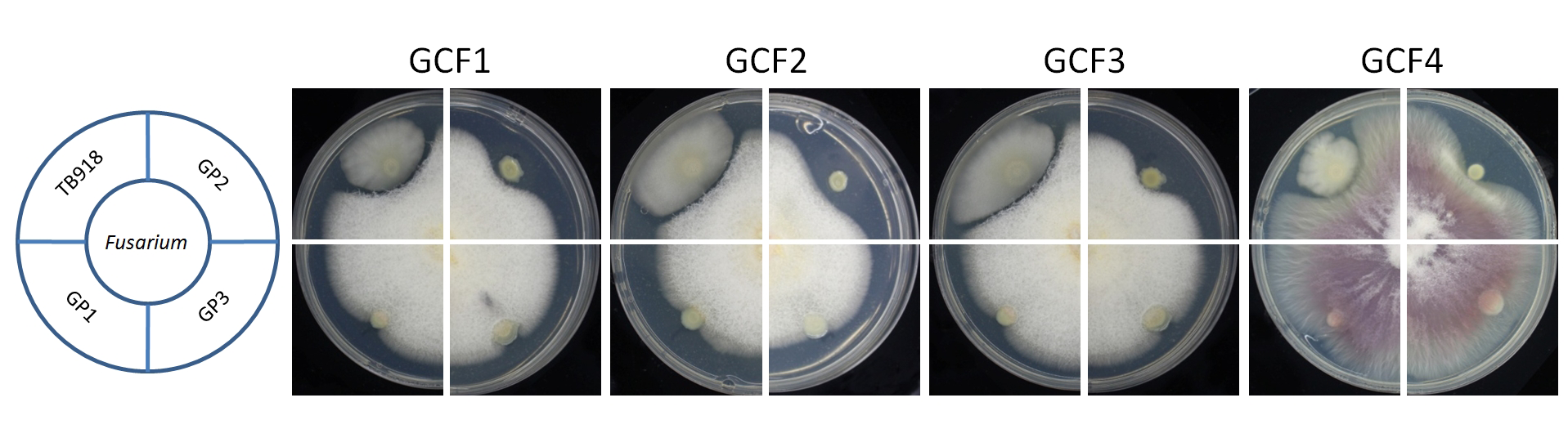

Supplement: Supplementary Figure S7 — Tablet antagonism of B. velezensis TB918 and three Pseudomonas isolates against four Fusarium isolates.The schematic diagram means: the center of each tablet is Fusarium isolates, the four quadrants are in order as P. fluorescens GP2, P. gessardii GP3, P. brenneri GP1 and B. velezensis TB918. Four Fusarium isolates are: F. solani GCF1, F. equiseti GCF2, F. solani GCF3, and F. proliferatum GCF4. [file Image_7.TIF]
